# The non-apoptotic function of Caspase-8 in negatively regulating the CDK9-mediated Ser2 phosphorylation of RNA polymerase II in cervical cancer

**DOI:** 10.1007/s00018-022-04598-3

**Published:** 2022-11-18

**Authors:** Ranadip Mandal, Monika Raab, Franz Rödel, Andrea Krämer, Izabela Kostova, Samuel Peña-Llopis, Gioele Medici, Björn Häupl, Thomas Oellerich, Khayal Gasimli, Mourad Sanhaji, Sven Becker, Klaus Strebhardt

**Affiliations:** 1 Department of Gynecology, Goethe University, University Hospital, Theodor-Stern-Kai 7, 60590 Frankfurt am Main, Germany; 2 Department of Radiotherapy and Oncology, University Hospital, Frankfurt am Main, Germany; 3 German Cancer Consortium (DKTK), Frankfurt am Main, Germany; 4 Frankfurt Cancer Institute (FCI), Goethe University Frankfurt, Frankfurt am Main, Germany; 5 Translational Genomics in Solid Tumors, West German Cancer Center, University Hospital, Essen, Germany; 6 German Cancer Consortium (DKTK), Essen, Germany; 7 German Cancer Research Center (DKFZ), Heidelberg, Germany; 8 Laboratory of Molecular Neuro-Oncology, Department of Neurology & Brain Tumor Center, University Hospital Zurich, University of Zurich, Zurich, Switzerland; 9 Clinical Neuroscience Center and Department of Neurosurgery, University Hospital Zurich, University of Zurich, Zurich, Switzerland; 10 Medizinische Klinik 2-Haematology/Oncology, University Hospital, Frankfurt am Main, Germany

**Keywords:** Caspase-8, Non-apoptotic function, Transcription, CDK9, P-TEFb, RNAPII

## Abstract

Cervical cancer is the fourth most frequently diagnosed and fatal gynecological cancer. 15–61% of all cases metastasize and develop chemoresistance, reducing the 5-year survival of cervical cancer patients to as low as 17%. Therefore, unraveling the mechanisms contributing to metastasis is critical in developing better-targeted therapies against it. Here, we have identified a novel mechanism where nuclear Caspase-8 directly interacts with and inhibits the activity of CDK9, thereby modulating RNAPII-mediated global transcription, including those of cell-migration- and cell-invasion-associated genes. Crucially, low Caspase-8 expression in cervical cancer patients leads to poor prognosis, higher CDK9 phosphorylation at Thr186, and increased RNAPII activity in cervical cancer cell lines and patient biopsies. Caspase-8 knock-out cells were also more resistant to the small-molecule CDK9 inhibitor BAY1251152 in both 2D- and 3D-culture conditions. Combining BAY1251152 with Cisplatin synergistically overcame chemoresistance of Caspase-8-deficient cervical cancer cells. Therefore, Caspase-8 expression could be a marker in chemoresistant cervical tumors, suggesting CDK9 inhibitor treatment for their sensitization to Cisplatin-based chemotherapy.

**Supplementary Information:**

The online version contains supplementary material available at 10.1007/s00018-022-04598-3.

## Introduction

Cervical cancer is the fourth most common gynecological cancer worldwide, accounting for 3.3% of all cancer-related deaths in 2018 [1]. Histologically, it is classified into Squamous Cell Carcinoma (SCC) (70%), adenocarcinoma (25%), and adeno-squamous carcinoma (3%). Rarer subtypes include neuro-endocrine or small-cell carcinomas. 99% of all cervical cancers are caused by persistent infections with the human papilloma virus (HPV) [2], whose early stages are usually asymptomatic. Only at later stages do symptoms arise, usually mistaken for other ailments. The development of Papanicolaou (PAP) smear screening and the HPV vaccine Gardasil has led to significant improvements in the early detection, treatment, and reduction of cervical cancer deaths, especially in developed countries. Treatment of cervical cancer involves surgery, radiotherapy, and platin-based chemotherapy (Carboplatin and Cisplatin), usually in combination, depending on the cancer stage. Nevertheless, 15–61% of all cases develop into metastatic disease [2], or drug resistance [3]. Also, ~ 33% of patients develop tumor recurrence within 18–24 years of initial diagnosis, depending on the tumor stage [4]. If detected early, cervical cancer has a 5-year survival rate of 92%; 56% if it has spread to the surrounding organs and/or lymph nodes; and only 17% when metastasized to distant organs [5]. Accordingly, a better understanding of molecular mechanisms like cell-migration and cell-invasion, critical for metastasis [6], is essential for developing novel treatment strategies against cervical cancer.

Pro-caspase-8 (hereafter Caspase-8) is a member of the Caspase family of cysteine–aspartate-specific proteases. It is the principal initiator of the extrinsic apoptotic pathway, besides being involved in intrinsic apoptosis, anoikis, autophagy, necroptosis, and pyroptosis [7–11]. The evasion of apoptosis is a hallmark of cancer and is observed in multiple cancer entities [10, 12, 13]. Mutations that inhibit Caspase-8’s activity are relatively rare. Instead, lesions accumulating in cancers (loss-of-function mutations of the pro-apoptotic *BAX* and *BAK1* genes, over-expressions of the anti-apoptotic Bcl-2 and Bcl-XL proteins, up-regulation of IAP family members, and post-translational modifications of Caspase-8) over their developmental phase, frequently interfere with the ability of Caspase-8 to execute apoptosis [10, 14–23]. Intriguingly, the down-regulation of Caspase-8’s activity has been shown to be beneficial for several cancers due to its involvement in non-apoptotic functions like cell-cycle regulation, proliferation, angiogenesis, and chemotherapy resistance [24–31]. For example, in neuroblastoma, head and neck squamous cell carcinomas, and triple-negative breast cancer, the down-regulation of Caspase-8 expression or its catalytic activity facilitates their cell-migration [10, 27–29]. However, the underlying molecular mechanisms are poorly understood.

Very limited information is available about the expression and functions of Caspase-8 in cervical cancer, although its activity decreases gradually as the malignancy progresses [21, 32]. Work in this direction has primarily attributed the enhanced metastasis or drug resistance in cervical cancer to the regulation of apoptosis or necroptosis in the presence or absence of Caspase-8 [32–37]. Therefore, understanding the non-apoptotic functions of Caspase-8 could help overcome cervical cancer metastasis and chemoresistance.

In the present study, we have demonstrated that Caspase-8 expression is frequently down-regulated in cervical cancer patients with high Tumor Mutational Burden (TMB), correlating significantly with poor prognosis. While investigating this mechanism, our interactome analysis revealed a novel interaction between Caspase-8 and CDK9. In cervical cancer cell lines, Caspase-8 negatively regulated the phosphorylation of CDK9 at Thr186 (pCDK9), thereby compromising its activation and the activity of the CDK9/CyclinT1 complex—P-TEFb (Positive Transcription Elongation Factor b) in phosphorylating the Ser2 residue at the C-terminal domain (CTD) of RNA polymerase II (RNAPII), thereby altering global transcription. The correlation between Caspase-8 expression and pCDK9 level was also observed in a cohort of cervical cancer primary materials. Knock-out and RNAi of Caspase-8 expression in cervical cancer cell lines enhanced cell-migration and cell-invasion by altering the RNAPII-mediated transcription of genes that regulated these functions. Work on cervical cancer cell lines revealed that Caspase-8, located in their nuclear fractions, could directly interact with and inhibit the phosphorylation of CDK9 at Thr186. Furthermore, our translational work with the small-molecule CDK9 inhibitor BAY1251152 [38] revealed that combining it with Cisplatin synergistically enhanced the sensitivity of Caspase-8 depleted cervical cancer cells to Cisplatin under both 2D- and 3D-cell culture conditions.

To our knowledge, this is the first-time Caspase-8 has been implicated in regulating the transcription of genes involved in the cell-migration and cell-invasion and contributing to chemoresistance and poor prognosis of cervical cancer patients. This likely occurs by altering RNAPII-mediated transcription elongation through the negative regulation of CDK9 autophosphorylation and its activity toward endogenous substrates.

## Materials and methods

### Cell culture

The HeLa, OVCAR-3, and OVCAR-8 cell lines were obtained from ATCC and cultured as per their instructions. The SiHa cells were cultured as described previously [39].

### Antibodies, reagents, siRNAs, and plasmids

Antibodies and sources: CDK9, pCDK9, RNAPII, phospho-RNAPII, TGM2, Cyclin B1, Cyclin E1, and NUP98 (Cell Signaling Technology); CDC37, SPT5, Stomatin, PLK1, Cyclin A1 (Santa Cruz Biotechnology); BRD4, Cyclin T1 and GFP (Abcam); Caspase-8 (Enzo Life Sciences); β-Actin, pCDK9, Vimentin, and Flag (Sigma-Aldrich).

Reagents and sources: CellTiter-Blue Cell Viability assay and Caspase-Glo 3/7 assay (Promega); BrdU kit (Roche); Thymidine, 5-ethynyl uridine (EU), Azide-fluor 488 (Sigma-Aldrich); AnnexinV and 7AAD (BD); [γ-32P] ATP (3000Ci/mmol, Amersham Pharmacia); Trail, FasL (Enzo Life Sciences); PLA assay kit (Olink Biosciences); BAY1251152, Cisplatin, and Carboplatin (Selleckchem); BioCoat Matrigel invasion chamber (Corning); Migration chamber (Ibidi); RNeasy Plus kit (Qiagen), active GST-CDK9/Cyclin K (SRP5012, Sigma-Aldrich).

The following vectors were used: pCas9(BB)-2A-Puro (PX459) V2.0 (62988, Addgene); p3xFlag-CMV-7.1 (E7533, Sigma); pGEX-5X-3 (28–9545-55, GE Healthcare) and pEGFP-C2 (6083-1, Clontech). All siRNAs and primers were from Sigma-Aldrich.

### CRISPR/Cas9-mediated stable knock-out of *CASP8*

As described earlier [17], the expression of the *CASP8* gene was stably knocked-out in the HeLa cells by targeting two distinct regions within its Exon 1, using the (PX459) plasmid. The target sequences were: (1) GCCTGGACTACATTCCGCAAAGG and (2) GCTCTTCCGAATTAATAGACTGG. Positive clones were confirmed through sequencing.

### In vitro kinase assays

Both radioactive and non-radioactive in vitro kinase assays were performed as described previously [40].

### Proximity Ligation Assay (PLA)

The proximity ligation assay was performed per the manufacturer’s protocol and as described previously [18].

### Caspase-Glo 3/7 assay

The Caspase-Glo 3/7 assay was performed per the manufacturer’s protocol and as described previously [41].

### Generation of spheroids and measurement of spheroid volumes

The generation and treatment of 3D spheroids were performed as described earlier [42].

The areas of the spheroids were measured using the ImageJ program, from which their radii were calculated. These radii were then used to determine the spheroid volumes. The following formula was used (*A* = area of spheroid; *r* = radius of spheroid; *V* = volume of spheroid):

$$A=4\pi {r}^{2}$$

$$r=\sqrt{\frac{A}{4\pi }}$$

$$V=\frac{4}{3}\pi {r}^{3}$$

### Phenotypic analysis

Cell-proliferation was measured using an CellTiter-Blue Cell Viability assay, as described earlier [41]. Briefly, the cells were seeded in 96-well flat-bottom plates, in 100 µl medium, for 24, 48, 72, 96, and 120 h. At each time point, 20 µl Resazurin reagent was added to each well, incubated at 37 °C for 3 h, and the fluorescence measured at 562/615 nm (Excitation/Emission).

Cell-cycle distribution was measured using PI-FACS measurement as described earlier [20].

2D cell-migration was assessed as described earlier [43]. Briefly, 0.1 × 10^6^ cells were seeded in 70 µl medium on each side of an ibidi migration chamber and allowed to settle-down overnight. For each cell type, 3 chambers were seeded per well of a 6-well plate. The chambers were then removed, the wells flooded with medium, and the migration of the cells, between the two cell populations from each chamber, was analyzed using time-lapse brightfield microscopy. The reductions in the areas between the two populations at each time point were quantified.

3D cell invasion was assessed as described earlier [43]. Briefly, 5 × 10^4^ cells were seeded in Matrigel-coated invasion chambers in a serum-starved medium. The chambers were placed in 12-well plates, also filled with serum-starved medium. Following overnight culture, only the medium in the wells was replaced with a serum-containing medium to allow the cells from the chambers to migrate toward this medium, through the Matrigel, for 24 h. The invaded cells were fixed and permeabilized, and their nuclei were stained with DAPI to be observed with fluorescence microscopy.

### 5-bromo-2′-deoxyuridine (BrdU) assay

The BrdU assay was performed as described previously [44] and per the manufacturer’s protocol. Briefly, HeLa WT or KO cells were first synchronized with a double-thymidine block in a 96-well plate. Following the second thymidine treatment, the cells were released into a fresh medium for 0, 3, 6, and 9 h. 2 h before each time point (except 0 h), 10 µM BrdU-labeling solution was added to the wells. Next, the labeling solution was removed, and the cells were dried at 60 °C for 1 h. After denaturing the DNA of the dried cells with the kit-supplied FixDenat solution, 100 µl of an anti-BrdU antibody was added to the wells for 1.5 h at 25 °C. Finally, the antibody was removed, 100 µl of the kit-supplied substrate solution was added to the wells, and their absorbance was measured after 5 min at 370 nm.

### 5-ethynyl uridine (EU) assay

The EU assay was performed as described previously [45]. Briefly, cells were seeded onto coverslips and incubated overnight. The growth medium was then replaced with a fresh medium including 500 µM 5-ethynyl uridine (EU) and incubated for 24 h. The cells were then fixed, permeabilized, and stained with a staining solution containing 10 µM Azide-fluor 488 for 30 min. Finally, the cells were stained with DAPI before being observed with fluorescence microscopy.

### Cytosol and nuclear fractionation

The cytosol and nuclear fractionation were performed, as mentioned earlier [46]. Briefly, harvested cells were incubated in Buffer A (10 mM HEPES, 10 mM KCl, 1.5 mM MgCL_2_, 0.34 M Sucrose, 10% glycerol, 1 mM DTT, protease inhibitor cocktail, and 0.1% Triton X-100) for 5 min. on ice and centrifuged at 1500×*g*. Cytosolic fraction was present in the supernatant. The nuclear fraction was obtained from the pellet by lysing with Buffer B (3 mM EDTA, 0.2 mM EGTA, 1 mM DTT, and protease inhibitor cocktail) for 10 min. on ice and centrifuging at 2000×*g*.

### RNA extraction and qRT-PCR

Total RNAs of cells were extracted with the EXTRACTME Total RNA Kit (Blirt), and their reverse transcription was performed using the GoScript Reverse Transcription Kit (Promega), as per the manufacturers’ instructions. All probes used for the quantitation of mRNA expression were obtained from Applied Biosystems: *GAPDH* (Hs02758991_g1), *Vimentin* (Hs00958111_m1), and *E-Cadherin* (Hs01023895_m1).

### Transcriptomics analysis

RNA from non-synchronized (NS) and S/G2-phase synchronized (synch.) HeLa WT and KO cells were extracted using the RNeasy Plus kit (Qiagen) and subjected to microarray expression profiling using the HumanHT-12 v3 arrays (Illumina, San Diego, USA) at the Microarray Unit of DKFZ (Heidelberg, Germany).

### Proteomics analysis

Global protein expression profiling was conducted as described before [47]. Briefly, non-synchronized (NS) and S/G2-phase synchronized (synch.) HeLa WT and KO cells were suspended in 500 µl lysis buffer [1% SDS/50 mM HEPES (pH 8.0) (with protease and phosphatase inhibitors)] and sonicated. After precipitation from the cleared lysates with acetone, the dried protein pellets were suspended in solubilization buffer (9 M urea, 20 mM HEPES, pH 8.0, 1 mM sodium orthovanadate, 2.5 mM sodium pyrophosphate, 1 mM beta-glycerophosphate), reduced with DTT and alkylated with iodoacetamide. Next, the protein samples were digested with Lys-C (Wako Chemicals) and trypsin (Promega), followed by a C18 peptide clean-up. Tandem Mass Tag (TMT) labeling was carried out according to the manufacturer’s instructions (Thermo Fisher Scientific), and the peptide mixtures were combined in a multiplexed sample, including one TMT channel containing a reference sample consisting of equal peptide amounts from each sample. After high-pH C18 RP kit pre-fractionation (Thermo Fisher Scientific), the samples were subjected to LC–MS/MS analysis on a Q Exactive HF Orbitrap mass spectrometer (Thermo Fisher Scientific) coupled to an UltiMate 3000 RSLCnano HPLC system (Dionex). After clean-up and concentration on a pre-column (ReproSil-Pur 120 C18-AQ (5 µm); Dr. Maisch GmbH), the samples were separated on an analytical column (ReproSil-Pur 120 C18-AQ (1.9 µm); Dr. Maisch GmbH).

### Interactome analysis

To identify Caspase-8 interacting proteins, the interactome analysis was performed as described earlier [48]. Essentially, Caspase-8 was immunoprecipitated (IP) from non-synchronized (NS) and S/G2-phase synchronized (synch.) HeLa WT cells and the co-immunoprecipitates (Co-IP) were loaded in a 12% SDS-PAGE gel and stained with Coomassie blue. IP from non-synchronized (NS) and S/G2-phase synchronized (synch.) HeLa KO cells were used as negative control (NC-1). IgG control was also included as an additional negative control (NC-2). After Coomassie staining, the lanes in the gel were excised into individual fragments and prepared for liquid chromatography-mass spectrometric (LC–MS) analysis.

The analysis of the Caspase-8 co-IP from the HeLa WT cells initially revealed a list of  ~ 6200 proteins. After ruling out any proteins which were also identified in NC-1 and NC-2, we narrowed down this list to  ~ 4000 proteins. Using a Log2 FC (Fold Change) cut-off of  ≥  ± 0.5,  ~ 1000 and  ~ 850 proteins were identified in the non-synchronized (NS) and S/G2-phase synchronized (synch.) data sets, respectively.

### Patient materials

For IHC staining, a total of 69 patients with uterine cervix squamous cell carcinoma treated with definitive chemoradiotherapy/brachytherapy (CRT/BT) at the Department of Radiotherapy and Oncology of the University Hospital Frankfurt, who provided informed consent and after institutional review board approval, were included in the study. Eligibility criteria covered histological proof of cervix carcinoma FIGO stages Ib to IVb. The median age was 59 years (range 21–89 years).

For RNAi experiments, primary cervical cancer samples were obtained from patients diagnosed and treated at the University Hospital Frankfurt. Cervical cancer cells were isolated from these samples, as mentioned previously [49].

### Immunohistochemical (IHC) staining and scoring

Formalin-fixed, paraffin-embedded (FFPE) pre-treatment biopsies were subjected to an HRP technique (DAKO Envision Flex, Hamburg, Germany) [50] with Caspase-8 (Thermo Fisher Scientific) and pCDK9 (Sigma) antibodies at 1:150 and 1:50 dilutions, respectively. Next, dextran-polymer-conjugated horseradish peroxidase and 3,3′-diaminobenzidine (DAB) chromogen were used for visualization and hematoxylin solution for counterstaining. Negative control slides in the absence of primary antibodies were included for each staining procedure. Two investigators (F.R., I.K.), blinded to patient clinical information, performed the evaluation to minimize inter-observer variability. Marker expressions were dichotomized as “high” [Weighted Score (WS) > 6] or “low” (≤ 6) based on a combination of the fraction of positive cells [1 (0–25%), 2 (26–50%), 3 (51–75%) and 4 (> 75%)] and the staining intensities [1 + (weak), 2 + (moderate) and 3 + (intense)] [16]. Image acquisition was performed using an AxioScanZ1 slide scanner and Zen software (Zeiss, Jena, Germany).

### Statistical analysis

For The Cancer Genome Atlas (TCGA) analysis, RNA-Seq and associated clinical data for CESC (cervical squamous cell carcinoma and endocervical adenocarcinoma) patients were obtained from the TCGA database (https://portal.gdc.cancer.gov/) and analyzed as described elsewhere [51]. Briefly, the RNA-Seq Expectation–Maximization (RSEM) normalized gene expressions for *CASP8* for the CESC-TCGA patients were stratified by quartiles, where the 1st quartile represented the lowest, 2nd and 3rd quartiles, the intermediate and 4th quartile the highest expressions. Overall Survival (OS) was computed by considering the patient’s date of death or the last follow-up date. Progression-Free Survival (PFS) was considered when distant metastasis or the patient’s death occurred. Tumor Mutational Burden (TMB) was obtained from TCGA Research Network [52]. Kaplan–Meier survival curves and log-rank tests were calculated with IBM SPSS Statistics v.25 (IBM, Ehningen, Germany).

Illumina arrays were quantile normalized and analyzed for the transcriptomics analysis as previously described [53]. Briefly, groups were compared by *t* tests adjusting for unequal variances and correcting for multiple testing.

Raw MS data were processed using the MaxQuant software (version 1.6.5.0, MPI for Biochemistry) [54]. Using the Andromeda search engine, fragment ion mass spectra were searched against the UniProt human reference proteome supplemented with 245 frequently observed contaminants [55]. The FDR was set to 1% on a decoy database approach at both the peptide and protein levels. Further data was evaluated using the Perseus software (v.1.6.0.7, MPI for Biochemistry) [56]. Potential contaminants, hits to the decoy database, and proteins identified solely with modified peptides were removed. Proteins identified with less than 2 unique peptides were excluded from further analysis. The TMT reporter intensities within each LC/MS run were normalized for equal sample loading and individual measurements by scaling to an internal reference containing equal peptide amounts of each sample [57].

The Combination Index (CI) was calculated based on the cell-proliferation and colony-formation assay results, using the Chou–Talalay method, as described earlier [58], and the Compusyn v.1 software, according to the developers’ instructions and as described earlier [59].

For the IHC staining, the correlation between pCDK9 expression, Caspase-8 levels, and pathological factors was assessed by computing Spearman’s correlation coefficient. *p* < 0.05 was considered statistically significant in all testing. IBM SPSS Statistics v.25 was used for the statistical analyses.

## Results

### Low Caspase-8 expression correlates with poor prognosis in cervical cancer patients

**Fig. 1:**
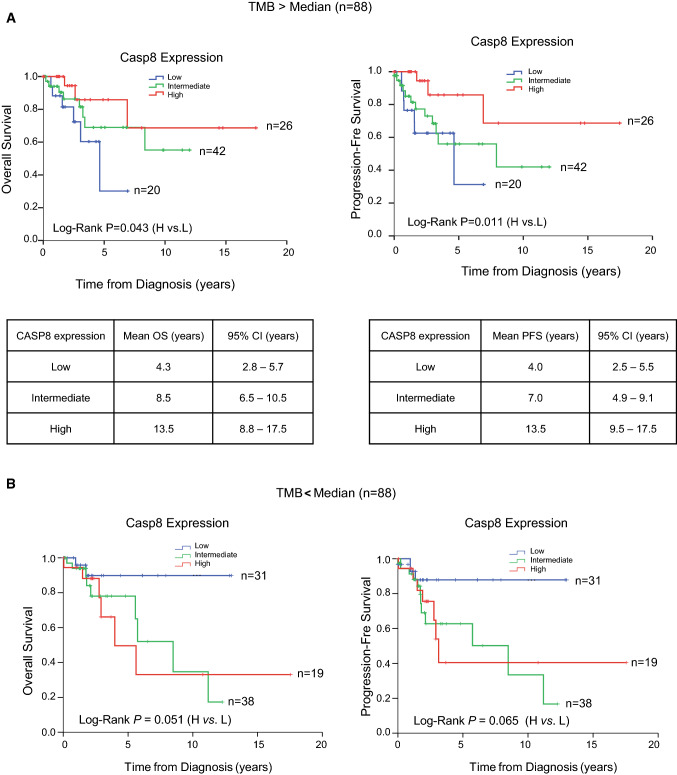
The effect of Caspase-8 expression on patient prognosis. **A** RNA-Seq data of cervical cancer patients with higher than median Tumor Mutational Burden (TMB) were obtained from the TCGA database (CESC-TCGA) and used to determine the correlation between high or low Caspase-8 expression with the Overall Survival (OS) and Progression-Free Survival (PFS) of 88 patients (upper panel). The tables show the mean OS and PFS of patients with low, intermediate, or high Caspase-8 expression, along with their 95% Confidence Interval (CI). **B** Correlation between high or low Caspase-8 expression with the OS and PFS of 88 patients with lower than median TMB based on their RNA-Seq data obtained from the CESC-TCGA database

Cervical cancer possesses one of the highest Tumor Mutational Burden (TMB) of all cancers [60]. To determine the significance of Caspase-8 expression in the prognosis of cervical cancer patients, we splitted the patients from the CESC-TCGA database, based on their RNA-Seq data, into those with TMB lower or higher than the median. Surprisingly, patients with high non-synonymous somatic mutations and low *CASP8* expression displayed significantly poorer OS (Overall Survival) and PFS (Progression-Free Survival) (Fig. 1A). In contrast, patients with TMB lower than the median, high *CASP8* expression displayed significantly poorer OS and PFS (Fig. 1B).

In summary, these data highlighted that the correlation between low *CASP8* expression and the prognosis of cervical cancer patients depends on their TMB status.

### Knock-out of Caspase-8 alters cellular behaviors

**Fig. 2:**
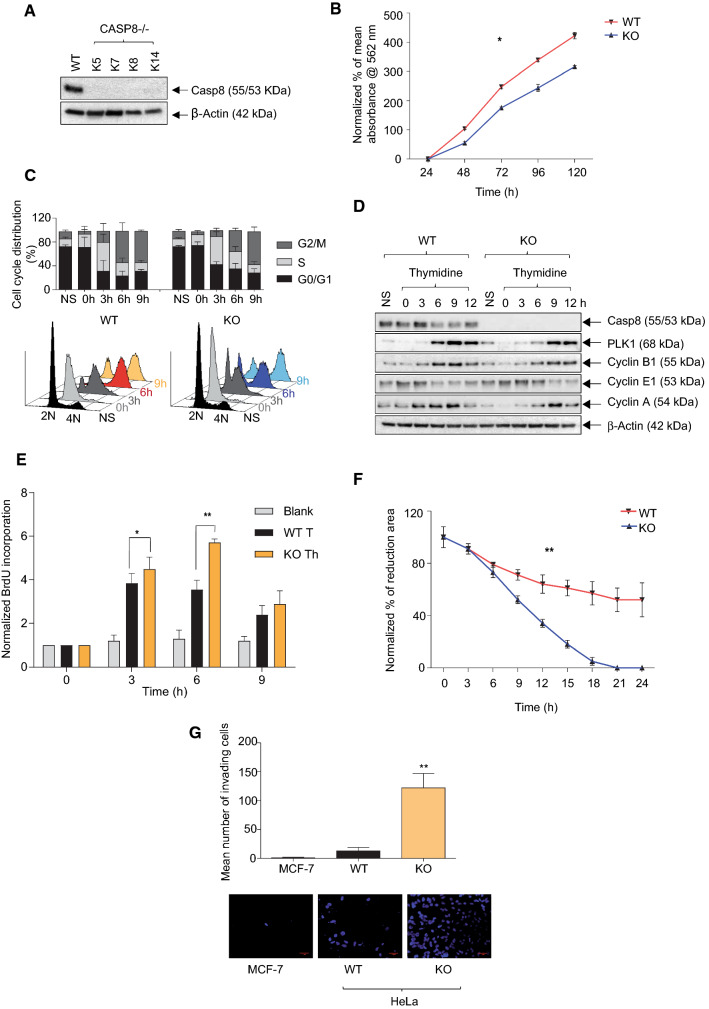
Effects of the knock-out of Caspase-8 on the behavior of cervical cancer cell lines. **A** HeLa *CASP8-/-* knock-out clones were generated using the CRISPR/Cas9 genome editing system. Individual clones (K5, 7, 8, and 14) were lysed and subjected to a Western blot analysis using Caspase-8 and β-Actin antibodies. **B** The proliferation of HeLa WT and KO cells was measured using an CellTiter-Blue Cell Viability assay. Over a period of 120 h, the number of viable cells were quantified every 24 h and represented graphically [mean ± SD; *n* = 3 for each time point; *p* value (paired *t* test, two-tailed); * =  < 0.05]. **C** For cell-cycle analysis, HeLa WT and KO cells were synchronized by double-thymidine treatment for 16 h and then released for 0, 3, 6, and 9 h. Cells were harvested, permeabilized with ethanol, and treated with Propidium Iodide (PI). After RNase treatment, the DNA histograms were determined by FACS to reveal the cell-cycle distribution of the cells at each time point. Non-synchronized (NS) WT and KO cells were used as negative controls. Overlay of the histograms of PI-positive WT and KO cells at every time point has been displayed in the lower panel; **D** lysates of synchronized cells were analyzed by immunoblotting to check for cell-cycle markers PLK1, Cyclins B1, E1, and A1, as well as Caspase-8 and β-Actin. **E** For the BrdU assay, HeLa WT and KO cells were first synchronized with double-thymidine for 16 h and released in a fresh medium for 0, 3, 6, and 9 h. 10 µM of the thymidine analogue BrdU was added to the cells for 2 h (before each time point, except 0 h) to allow it to be incorporated into the newly synthesized DNA and was detected using an anti-BrdU antibody. The absorbance, measured at 370 nm, correlating with the amount of incorporated BrdU into the newly synthesized DNA, has been represented graphically. The Blank represents the fluorescence intensity of the anti-BrdU antibody, without the BrdU agent [mean ± SD; *n* = 3 for each time point; *p* value (paired *t* test, two-tailed); * =  < 0.05; ** =  < 0.005]. **F** The 2D migration of HeLa WT and KO cells was determined using ibidi migration chambers at 3 h intervals over 24 h. The reductions in the areas between the two cell populations at each time point, representing the migration of the cells, were measured, normalized to the area at 0 h, and represented graphically [mean ± SD; *n* = 3 for each time point; *p* value (paired *t* test, two-tailed); ** =  < 0.005]. **G** The 3D invasion of HeLa WT and KO cells was determined using Matrigel-coated invasion chambers was determined over a period of 24 h. The nuclei of the invaded cells were stained with DAPI (bottom panel), and their quantification was represented graphically. MCF-7 cells were used as a negative control [mean ± SD; *n* = 3 for each time point; *p* value (paired *t* test, two-tailed); ** =  < 0.005]

To further explore the roles of Caspase-8 expression in the malignant behavior of cervical cancer cells that might contribute to poor patient prognosis, we used the CRISPR/Cas9 system to generate *CASP8-/-* HeLa and SiHa cells (Fig. 2A, Supplementary Fig. 1A). To avoid clonal variations [61], we mixed individual *CASP8-/-* knock-out clones—Clones K5, 7 and 8 for HeLa and Clones 7, 11 and 22 for SiHa, to form a mixed knock-out population (henceforth KO) and determined different biological traits of the resulting cells. We first determined the 2D cell-proliferation of the WT vs. KO cells over 120 h and observed that the KO HeLa cells proliferated at a significantly slower rate than the WT cells (Fig. 2B). Importantly, none of the individual knock-out clones displayed any significant differences in their cell-proliferations (Supplementary Fig. 2A), indicating that none of the individual clones significantly affected the differential proliferation of the KO population.

We next decided to determine whether the reduced proliferative activity of KO cells could be attributed to any alterations in their cell-cycle profiles. For this, HeLa and SiHa WT and KO cells were synchronized with a double-thymidine block, released into a fresh medium, and analyzed by FACS. We observed that the cell-cycle profile of synchronized KO cells was retarded primarily at the 6 h time point, which corresponded with the S/G2-phase of the cell-cycle, compared to that of WT cells, albeit more prominently in HeLa than in SiHa cells (Fig. 2C, Supplementary Fig. 1B). The expression of cell-cycle markers for G1/S (Cyclin E1), S/G2 (Cyclin A1) and G2/M (PLK1, Cyclin B1) confirmed this cell-cycle delay in the KO cells of both cell lines (Fig. 2D, Supplementary Fig. 1C). Furthermore, the significant increase in the BrdU signals, which relies on the incorporation of the thymidine analogue 5-bromo-2′-deoxyuridine (BrdU) into the nascently synthesized DNA strands [44], after 3 and 6 h release from double-thymidine synchronization, confirmed the cell-cycle delay observed in the HeLa KO cells (Fig. 2E).

Moreover, comparing the 2D migration and 3D invasion between the KO and WT cells demonstrated that KO cells migrated (Fig. 2F, Supplementary Fig. 1D) and invaded (Fig. 2G, Supplementary Fig. 1E) at significantly faster rates than their WT counterparts. Importantly, none of the individual knock-out clones displayed any significant differences in their cell-migrations (Supplementary Fig. 2B), indicating that none of the individual clones significantly affected the differential migration of the KO population.

Knocking-down Caspase-8 expression using siCasp8 in HeLa and SiHa WT cells (Supplementary Fig. 3A) significantly enhanced their invasiveness as compared to Empty Flag-Vector (EV) transfected or non-transfected (NT) cells (Supplementary Fig. 3B). Reversibly, over-expressing a Flag-tagged-Casp8 (Flag-Casp8) in HeLa WT and KO cells significantly reduced invasion (except WT Flag-Casp8 vs. WT Empty Flag-Vector) and migration, as compared to their respective Empty Flag-Vector (EV) counterparts (Supplementary Figs. 3B, C).

In summary, these experiments demonstrated that the loss of Caspase-8 expression in HeLa and SiHa cells reduced cell-proliferation, which could be attributed to a delay in the cell-cycle progression at the S/G2 phase. In addition, the loss of Caspase-8 expression caused a significant enhancement of the 2D-migration and 3D-invasion of both cell lines. Importantly, we observed no significant increase in apoptosis in the KO cells, even after treating them with Trail (Supplementary Fig. 3D), further validating the veracity of our Caspase-8 knock-out.

### CDK9 is an interacting partner of Caspase-8

The results of the previous experiments raised a fundamental question on how Caspase-8 alters the migration and invasion of cervical cancer cells. To elucidate this, we IPed Caspase-8 from non-synchronized (NS) exponentially growing and S/G2-phase synchronized (synch.) HeLa WT. The co-IPed proteins were subjected to an interactome analysis to identify Caspase-8 interacting proteins that might be involved in cell-migration and cell-invasion.

After performing the initial analysis of the non-synchronized (NS, ~ 1000 proteins) and S/G2-phase synchronized (synch., ~ 850 proteins) data sets, we compared the ratios of their Log2 FC ≥  ± 0.5 values (up- and down-regulated) to obtain 551 common proteins between the NS and synch. data sets (Supplementary Figs. 4A, B). Finally, we selected 291 proteins for further analysis, which were present in the non-synchronized (NS) and S/G2-phase synchronized (synch.) data sets, with a Log2 FC ≥  + 0.5 (up-regulated), representing the pool of potential Caspase-8 interacting proteins (Supplementary Fig. 4C). Expectedly, Caspase-8 had one of the highest Log2 FC values in the synchronized (NS) and S/G2-phase synchronized (synch.) data sets (Supplementary Fig. 4C). The step-by-step analysis of our interactome study, to obtain the 291 proteins, has been schematically represented here (Supplementary Fig. 4D).

**Fig. 3:**
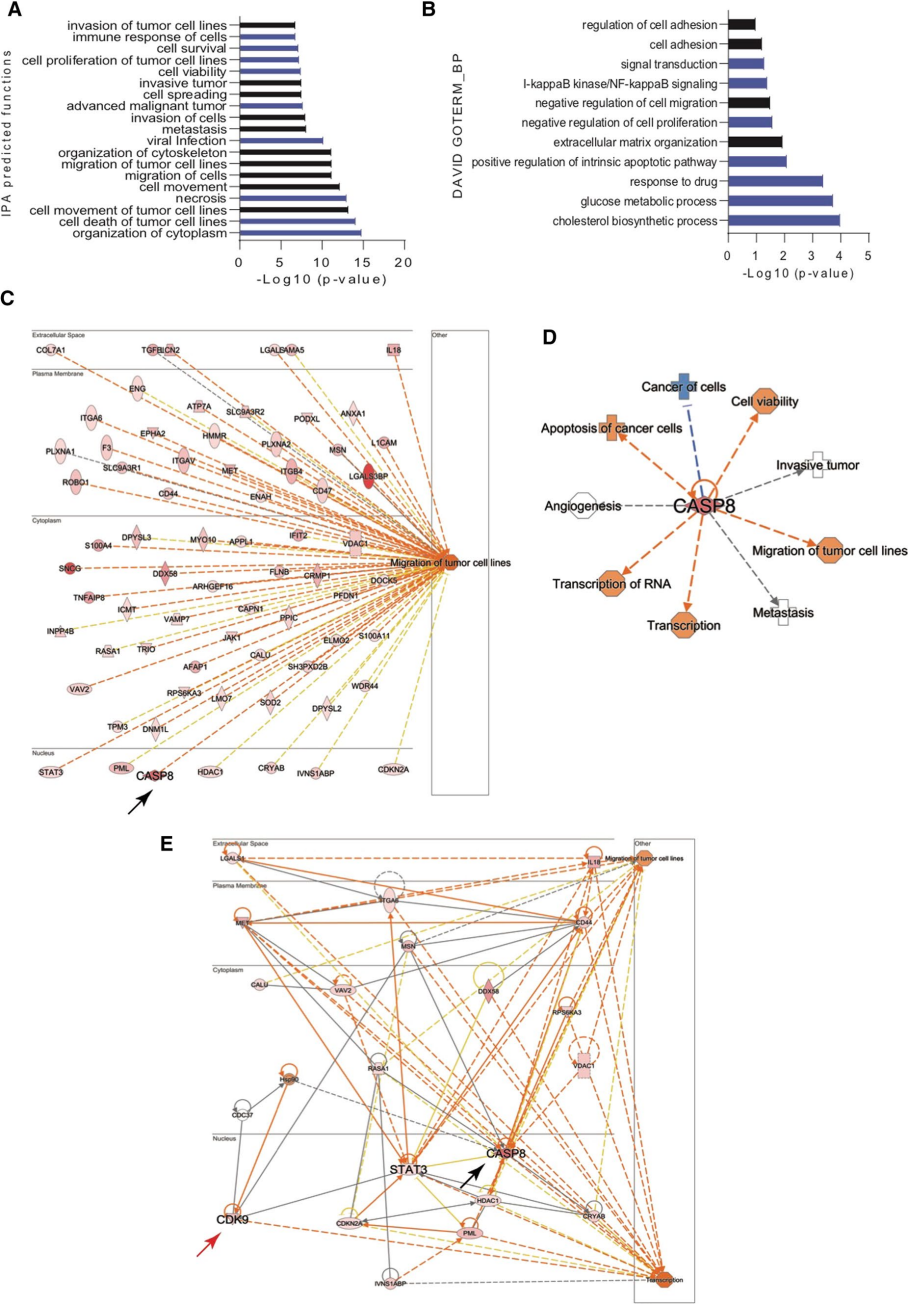
Caspase-8 interactome. 291 differentially expressed proteins with a Log2 FC ≥  + 0.5 (up-regulated), representing the pool of potentially Caspase-8 interacting proteins, were identified through the comparative analysis of HeLa WT non-synchronized (NS) and S/G2-phase synchronized (synch.) data sets. They were analyzed for their cellular functions using **A** the Ingenuity Pathway Analysis (IPA) bioinformatics tool. The top 20 most significantly regulated pathways are shown here, predicted by IPA. The black bars represent cancer cell-migration associated functions; and **B** the DAVID bioinformatics tool (www.david.ncifcrf.gov, v.6.8). Shown here are the top 15 most significant processes, predicted using the Gene Ontology Term (GOTERM) “Biological Process (BP)” function of DAVID. The black bars represent cancer cell-migration-associated processes. **C** The 291 proteins predicted by IPA to be associated with cell-migration were further analyzed by IPA to determine whether these proteins, including Caspase-8, were directly involved with cell-migration or indirectly, via other proteins, which may or may not be present in our data-set of 291 proteins. The black arrow highlights Caspase-8. **D** The “Diseases and Functions” feature of the IPA tool also predicted that the cell-migration-associated proteins were also involved in several other biological processes. Shown here are the top 10 processes regulated by these proteins and their associations with Caspase-8. **E** The proteins involved in both cell-migration and transcription were further analyzed using IPA to determine whether Caspase-8 could directly or indirectly interact with them. The black and red arrows highlight Caspase-8 and CDK9, respectively. The interactome analysis was performed in triplicate

We next investigated the molecular networks/pathways in which these 291 proteins participate and the potential involvement of Caspase-8 in them using Ingenuity Pathway Analysis (IPA). Intriguingly, IPA analysis revealed a wide variety of molecular pathways critical for the growth and survival of tumor cells, including those involved in tumor cell-migration (Fig. 3A, highlighted in black). Furthermore, GOTERM_BP (Biological Process) analysis using the DAVID bioinformatics tool revealed several cell-migration-associated biological functions (Fig. 3B, highlighted in black).

We next used IPA to perform their network analysis to elucidate the correlation of Caspase-8 with proteins involved in these pathways. Interestingly, IPA predicted that Caspase-8 is not only directly involved with the cell-migration pathway (Fig. 3C, black arrow), whose constituent proteins, including Caspase-8, appear to form a tight cluster (Supplementary Fig. 4E, black arrow), but also with several other biological processes, including transcription (Fig. 3D). This data encouraged us to investigate potential correlations between cell-migration and transcription-associated proteins. Notably, IPA identified the kinase CDK9, which was involved in both the pathways, to interact with Caspase-8, albeit indirectly via the transcription factor STAT3 and the CDC37/HSP90 chaperone complex (Fig. 3E, red arrow).

In summary, the interactome analysis of Caspase-8 revealed that it could potentially interact with a wide variety of proteins, including CDK9, regulating different molecular pathways, including those involved in cell-migration. Surprisingly, in both analyses, IPA predicted Caspase-8 as a nuclear protein (Fig. 3C, E,black arrow), even though it is generally known to be a cytoplasmic protein [10, 17]. Based on these predictions and the fact that CDK9 is a predominantly nuclear protein [38], we next aimed to study the potential interaction between Caspase-8 and CDK9.

**Fig. 4:**
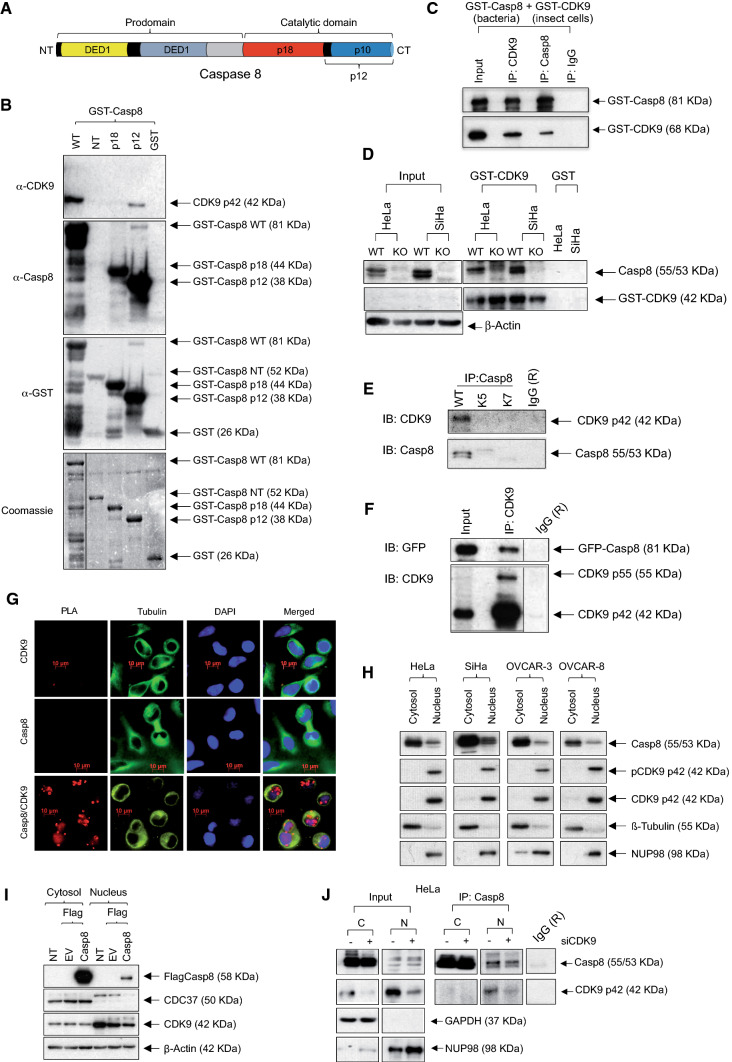
Interaction between Caspase-8 and CDK9. **A** Domain structure of the Caspase-8 protein. *NT* N-Terminus, *CT* C-Terminus and *DED* Death Effector Domain. **B** GST-tagged-Casp8 (full-length)-WT, -NT, -p18, and -p12 fragments were used to pull-down endogenous CDK9 from the lysates of HeLa WT cells. Pull-down analyses using an uncoupled GST protein (GST) were used as the negative control. An anti-CDK9 antibody was used to detect the presence of CDK9 in an immunoblot of the pull-down assay, whereas anti-Caspase-8 and -GST antibodies were used to identify/confirm the presence of the GST-Caspase-8 (WT and fragments)/GST bait proteins in the same immunoblot. Coomassie staining was performed to determine the expressions of the different GST-tagged proteins (lowest panel). **C **Commercially available, recombinant GST-CDK9 of Baculovirus origin was incubated with recombinant GST-Casp8-WT of bacterial origin. CDK9 and Caspase-8 specific antibodies were used to immunoprecipitate GST-CDK9 and GST-Casp8-WT, respectively. Input and IgG controls have also been included. **D** GST-tagged-CDK9 (GST-CDK9) was used to pull-down endogenous Caspase-8 from lysates of HeLa and SiHa (WT, KO) cells. An immunoblot of the lysate input was probed for Caspase-8 and β-Actin (left panel), whereas the pull-down was probed for Caspase-8 and GST-CDK9 bait protein (right panel). Pull-downs from HeLa and SiHa WT lysates, using GST, were included as negative controls. **E** A specific antibody was used to precipitate Caspase-8 from the lysates of WT and two knock-out clones (K5, K7) of HeLa cells and probed for CDK9 and Caspase-8 in an immunoblot (IB). **F** A GFP-tagged-Caspase-8 (GFP-Casp8) was first expressed in HeLa WT cells and then used to perform a CDK9 IP. Immunoblot of the lysate input (left lane), CDK9 IP (center lane), and IgG control (right lane) were probed for Caspase-8 and CDK9. **G** A Proximity Ligation Assay (PLA) was performed by staining HeLa WT cells using anti-CDK9 or -Casp8 antibodies individually or together. The cells were then stained with fluorescent-tagged PLA probes. PLA signals (red spots) were observed under a fluorescence microscope. DAPI and Tubulin staining were used to highlight the nucleus and cytoplasm of the cells, respectively. **H** The cervical cancer cell lines (HeLa) and the high-grade serous ovarian cancer cell lines (OVCAR-3, OVCAR-8) were fractionated into their respective cytosol and nuclear fractions. These fractions were immunoblotted to check for Caspase-8, pCDK9, CDK9, β-Tubulin (cytosolic marker), and NUP98 (nuclear marker). **I** HeLa KO cells expressing either an Empty Flag-Vector (EV, negative control) or Flag-tagged-Caspase-8 (Flag-Casp8) were similarly fractionated into their cytosol and nuclear fractions. Identically fractionated non-transfected (NT) KO cells were also included. All cytosol and nuclear fractions were immunoblotted to check for Flag-Caspase-8, CDK9, CDC37 (cytosolic marker), and β-Actin (whole-cell marker). **J** Caspase-8 was IPed, using an anti-Caspase-8 antibody from the cytosol (C) and nuclear (N) fractions of HeLa WT siCtrl and CDK9-depleted cells (siCDK9). Lysate input, shown on the left side, and the IP on the right, were checked for Caspase-8 and CDK9, whereas the inputs were additionally checked for GAPDH (cytosolic marker) and NUP98. IgG (R) control has also been demonstrated

To achieve this goal, we first performed a ‘pull-down’ assay from the lysates of HeLa KO cells, using GST-Casp8-WT, -NT (N-terminal region/prodomain), -p18, and -p12 domains (making up its C-terminal region/catalytic domain) [10] (Fig. 4A) and observed that endogenous CDK9 interacted with the GST-Casp8-WT, specifically with the p12 domain (Fig. 4B). Since most cellular CDK9 is found either as part of the large negative regulatory complex—7SK snRNP, within the nucleus or with the CDC37/HSP90 chaperone complex, within the cytosol [38, 62], we next decided to identify whether the interaction between CDK9 and Caspase-8 occurred directly, or via any intermediate proteins, which could be a part of these complexes. For this purpose, we incubated a commercially available, recombinant, active P-TEFb (GST-CDK9/Cyclin K) of Baculovirus origin with recombinant GST-Casp8-WT of bacterial origin in a cell-free system and performed immunoprecipitations using specific antibodies. IP with an anti-CDK9 antibody immunoprecipitated GST-CDK9 and co-immunoprecipitated the GST-Casp8-WT, whereas the IP with an anti-Caspase-8 antibody immunoprecipitated GST-Casp8-WT and co-immunoprecipitated the GST-CDK9. This confirmed that the interaction between the two proteins occurs directly, without the involvement of any intermediate bridging proteins (Fig. 4C).

Reversibly, GST-CDK9 also pulled down endogenous Caspase-8 in both HeLa and SiHa WT cells, but, expectedly, not from their respective KO cells (Fig. 4D). Furthermore, endogenous CDK9 was also co-IPed in a Caspase-8 IP of HeLa WT cells, but expectedly, not using two KO clones (Fig. 4E). Reversibly, exogenous GFP-Casp8, overexpressed in HeLa WT cells, was also co-IPed with CDK9 (Fig. 4F). A Proximity Ligation Assay (PLA) further showed a tremendous increase in red fluorescent spots in HeLa WT cells stained together with anti-CDK9 and -Caspase-8 antibodies, as compared to when they were used individually (Fig. 4G), indicating an interaction between CDK9 and Caspase-8 in living cells. In summary, these experiments confirmed that CDK9 directly interacts with Caspase-8 both in vitro and in cells.

However, the question remained about the cellular compartment where this interaction occurs. This is because CDK9 is predominantly nuclear, where it undergoes activation by phosphorylation at its Thr186 residue, promoting its binding to its cyclin partners, predominantly Cyclin T1, to form P-TEFb [38]. To verify IPA’s prediction of the nuclear presence of Caspase-8, we separated the lysates of HeLa, SiHa, and two High-Grade Serous Ovarian Cancer (HGSOC) cell lines—OVCAR-3 [63] and OVCAR-8 [64], into their cytosol and nuclear fractions. We observed that while endogenous Caspase-8 was predominantly cytoplasmic, it was present, at lower levels, within the nucleus of every cell line (Fig. 4H). Furthermore, similar fractionation of HeLa KO cells, over-expressing Flag-Casp8, also exhibited low expression of the exogenous Caspase-8 within the nucleus (Fig. 4I). To investigate whether Caspase-8 and CDK9 would interact within the nucleus, we IPed Caspase-8 from the cytosol and nucleus of HeLa WT cells, in which CDK9 expression was downregulated with siCDK9. Indeed, Caspase-8 co-IPed CDK9 only from the nuclear fraction of the siCtrl (-) cells (Fig. 4J).

In summary, we have confirmed that CDK9 interacts with the p12 domain of Caspase-8 within the nucleus of cervical cancer cells. We cannot rule out any potential interaction between Caspase-8 and CDK9 in the cytosol. However, at least under our experimental conditions, this interaction in the cytosol was below the limit of detection of the Caspase-8 IP (Fig. 4J).

### Caspase-8 inhibits the activity of CDK9

**Fig. 5:**
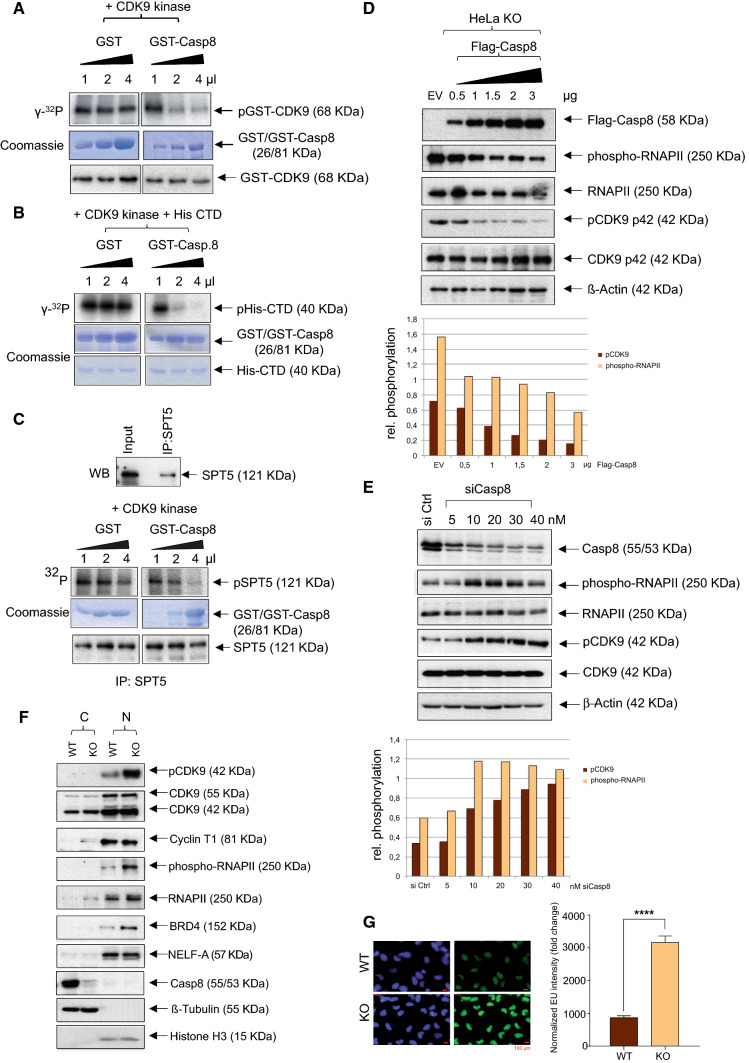
Effect of Caspase-8 on the autophosphorylation and enzymatic activity of CDK9. **A** For in vitro radioactive kinase assays increasing amounts of GST-Casp8 or GST were incubated with a commercially available, active GST-CDK9/Cyclin K and radioactive γ-^32^P-labeled ATP. The autoradiogram of phosphorylated GST-CDK9 (upper panel) and the Coomassie staining of GST-Casp8/GST (middle panel) are shown. Immunoblot of GST-CDK9 probed with a CDK9-specific antibody has been shown in the lower panel. **B** Increasing amounts of GST-Casp8 or GST were incubated with active GST-CDK9/Cyclin K along with His-tagged C-terminal domain (His-CTD) of RNAPII in a radioactive kinase assay (upper panel). Coomassie staining of GST-Casp8/GST (middle panel) and His-CTD (lower panel) is shown. **C** SPT5 was IPed from lysates of HeLa WT cells using a specific SPT5 antibody. The immunoblot of the IP and input control, which were probed for SPT, is shown (upper panel). The precipitated SPT5 protein was incubated with increasing amounts of GST-Casp8 or GST, active GST-CDK9/Cyclin K, and radioactive γ-^32^P labeled ATP (middle panel). The Coomassie staining of GST-Casp8/GST is depicted (lower panel). Immunoblot showing the levels of the precipitated, non-phosphorylated SPT5, used as the substrate in the kinase assay, probed with the SPT5 antibody, has been shown in the lowest panel. **D** HeLa KO cells were transfected with either an Empty Flag-Vector (EV, negative control) or increasing amounts of Flag-Casp8, immunoblotted, and checked for Flag-Casp8, phospho-RNAPII, RNAPII, pCDK9, CDK9, and β-Actin (upper panel). The levels of pCDK9 and phospho-RNAPII, in the presence of increasing amounts of Flag-Casp8, normalized to their respective CDK9 and RNAPII levels, are shown in a bar graph (lower panel). **E** HeLa WT cells were either transfected with siCtrl or with increasing concentrations of siCasp8, immunoblotted, and checked for Caspase-8, phospho-RNAPII, RNAPII, pCDK9, CDK9, and β-Actin (upper panel). The levels of pCDK9 and phospho-RNAPII, in the presence of increasing concentrations of siCasp8, normalized to their respective CDK9 and RNAPII levels, have been shown graphically (lower panel). **F** The cytosol (C) and nucleus (N) of HeLa WT and KO cells were fractionated, immunoblotted, and checked for pCDK9, CDK9, Cyclin T1, phospho-RNAPII, RNAPII, transcription regulators BRD4 and NELF-A, Caspase-8, β-Tubulin (cytosolic marker) and Histone H3 (nuclear marker). **G** HeLa WT and KO cells were treated with 5-ethynyl uridine (EU) or DAPI for staining nuclei (left panel). The ratio of fluorescence intensity of EU to DAPI stain was quantified, normalized, and graphically represented (right panel) [mean ± SD; *n* = 3; *p* value (paired *t* test, two-tailed); **** =  < 0.0001]

Having demonstrated the interaction between Caspase-8 and CDK9, the next question was about the effect of this interaction on each other. As CDK9 is a kinase, we first asked whether Caspase-8 may be a target for phosphorylation. Therefore, we performed an in vitro kinase assay with γ-^32^P ATP involving the active P-TEFb (GST-CDK9/Cyclin K) incubated with increasing amounts of GST-Casp8 or GST (Fig. 5A). Surprisingly, instead of P-TEFb phosphorylating Caspase-8, we observed a GST-Casp8 amount-dependent reduction of P-TEFb phosphorylation. Increasing amounts of GST, used as a control, did not influence the phosphorylation of P-TEFb (Fig. 5A, Supplementary Fig. 5A). Importantly, both GST-Caspase-8 and GST did not affect the levels of non-phosphorylated GST-CDK9, as determined by immunoblot (Fig. 5A, lower panel).

The principal cellular function of P-TEFb is to mediate the productive transcriptional elongation by phosphorylating the Ser2 residue of RNAPII [38]. As we have demonstrated that Caspase-8 impedes the phosphorylation of P-TEFb in vitro (Fig. 5A, Supplementary Fig. 5A), we next sought to investigate whether Caspase-8’s interaction with P-TEFb would affect its kinase activity toward RNAPII. With this aim, we performed a similar kinase assay but also included the carboxy-terminal domain (CTD) of RNAPII as a His-tagged recombinant protein (His-CTD). Intriguingly, the presence of GST-Caspase-8, but not GST, triggered an amount-dependent reduction in the phosphorylation of His-CTD (Fig. 5B, Supplementary Fig. 5B). These data suggested that Caspase-8’s interaction with CDK9 blocked P-TEFb’s kinase activity, which in turn affected the P-TEFb dependent phosphorylation of RNAPII.

Next, we were curious whether this inhibition of P-TEFb’s kinase activity also extended to other substrates. P-TEFb has been reported to phosphorylate the SPT5 subunit of DSIF [(DRB) Sensitivity-Inducing Factor], a negative regulator of transcription elongation [38]. As no commercial phsospho-specific antibodies against SPT5 exist, we IPed SPT5 from HeLa WT cells and performed a similar kinase assay with P-TEFb. In line with the previous results, we observed a GST-Casp8, but not GST, amount-dependent reduction in the phosphorylation of the IPed SPT5 by P-TEFb (Fig. 5C, Supplementary Fig. 5C). Once again, both GST-Caspase-8 and GST did not affect the endogenous levels of SPT5, as determined by immunoblot of the IPd SPT5 (Fig. 5C, lowest panel). In summary, we could confirm that in vitro, Caspase-8 inhibited the activity of P-TEFb toward at least two major substrates.

Our next objective was to specify whether this inhibition occurred at Thr186, the activation site of CDK9, and Ser2, CDK9’s phosphorylation site on RNAPII [38]. With this aim, we performed a non-radioactive kinase assay by first precipitating active CDK9, using a Cyclin T1 antibody from WT and two KO clones of HeLa cells, and incubating the precipitates with His-CTD and ATP. We observed higher levels of phospho-Ser2-His-CTD, using a specific phospho-RNAPII (Ser2) antibody, in both the KO clones as compared to the WT cells (Supplementary Fig. 6A). To verify whether Caspase-8 manifests these inhibitory effects within cells as well, we next over-expressed increasing amounts of Flag-Casp8 in HeLa KO cells and observed a Flag-Casp8 amount-dependent decrease in Ser2 phosphorylation of RNAPII (henceforth phospho-RNAPII) and Thr186 phosphorylation of CDK9 (henceforth pCDK9) (Fig. 5D, bar graph). On the other hand, down-regulating *CASP8* in HeLa WT cells showed a siCasp8 concentration-dependent increase in phospho-RNAPII and pCDK9 (Fig. 5E, bar graph). Additionally, the cytoplasm and nuclear fractions of WT and KO HeLa (Fig. 5F) and SiHa (Supplementary Fig. 6B) cells also showed increased levels of pCDK9 and phospho-RNAPII in the nuclei of the KO cells as compared to the WT cells.

In summary, these experiments have confirmed Caspase-8’s role in negatively regulating the Thr186 phosphorylation of CDK9 and its kinase activity on the Ser2 phosphorylation of RNAPII within cells.

Due to phospho-RNAPII’s role in transcriptional regulation, we next investigated whether Caspase-8 expression altered overall transcription in the cells. For this, we performed an EU assay, which relies upon incorporating the uridine analogue—5-ethynyl uridine (EU) into the nascently synthesized RNA [45]. Interestingly, we observed a significant increase in the EU uptake in the KO vs. WT cells, suggesting an alteration in overall transcription in the absence of Caspase-8 expression (Fig. 5G). To further confirm Caspase-8’s role in altering overall transcription, we repeated the EU assay by expressing increasing amounts of Flag-Casp8 or siCasp8 in the KO and WT cells, respectively. We observed a significant Flag-Casp8 amount-dependent reduction of EU uptake, as indicated by the decrease in fluorescent signal, as compared to Empty Flag-Vector (EV, negative control) (Supplementary Fig. 6C). In contrast, we observed a significant siCasp8 concentration-dependent increase in fluorescent signal, as compared to siCtrl transfected WT cells (Supplementary Fig. 6D).

In summary, the EU experiments suggested that the loss of Caspase-8 expression altered the overall transcription in HeLa and SiHa cells, possibly by increasing the phosphorylation of CDK9 at Thr186 and CDK9’s targeted phosphorylation of SPT5 and RNAPII.

### Caspase-8 inhibits the phosphorylation of CDK9 in primary cervical cancer samples

**Fig. 6:**
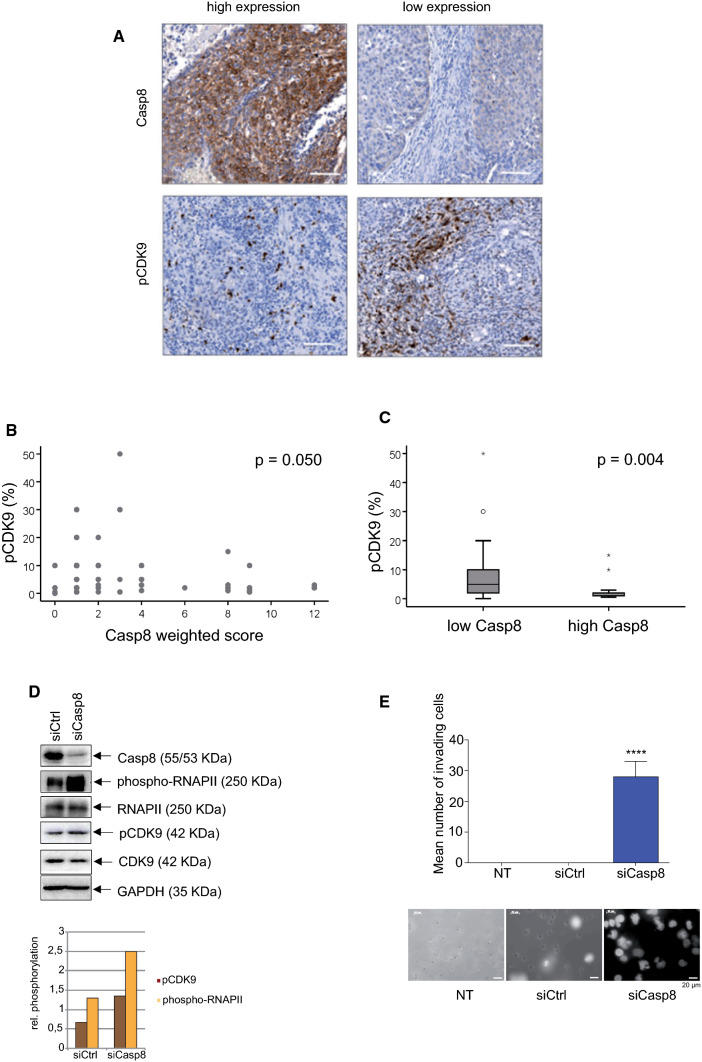
Correlation between Caspase-8 expression and pCDK9 in cervical cancer patients. **A** Examples of cervix cancer biopsies with high and low immunohistochemical detection of Caspase-8 and pCDK9 (Thr186). Original magnification × 100, scale bar: 100 μm. **B** The association of the immunohistochemical detection of pCDK9 (percentage of positive cells) and the Caspase-8 weighted score (intensity of staining × percent of positive tumor cells) in pretreatment biopsies of 69 patients. **C** The association of the immunohistochemical detection of pCDK9 with high (score 8–12) and low (score 0–6) Caspase-8 expression. The tick line is the median value, the solid box is the interquartile range, and the whiskers are the 5th and 95th percentiles. **D** Immunoblot of the lysate of a cervical cancer primary patient cells, in which Caspase-8 expression was either knocked down using siCasp8 or transfected with siCtrl. The immunoblot was checked for the levels of Caspase-8, phospho-RNAPII, RNAPII, pCDK9, CDK9, and GAPDH. The levels of pCDK9 and phospho-RNAPII in siCtrl and siCasp8 transfected cells, normalized to their respective CDK9 and RNAPII levels, have been shown graphically. **E** 3D cell-invasion assay of patient-derived cervical cancer primary cells transfected with either siCasp8 or siCtrl or was non-transfected (NT). Invaded cells were stained with DAPI, and their quantification was graphically represented. The bottom panel shows the fluorescent images of DAPI stained invaded cells of each type [*n* = 3; mean ± SD; *p* value (paired *t* test, two-tailed); **** =  < 0.0001]

Our work so far has demonstrated a direct correlation between Caspase-8 expression and pCDK9 levels in cervical cancer cell lines. However, we were curious whether this correlation also extended to primary cervical cancer tissues. To investigate this, we performed IHC staining of pre-treatment biopsies from 69 cervical cancer patients (FIGO IB–IVA) for Caspase-8 and pCDK9 (Fig. 6A). The median pCDK9 index for all patients was 2.0% (range of 0%—50%). The Weighted Score (WS) of Caspase-8 expression revealed a significant correlation with pCDK9 levels in corresponding tumor tissues (*p* = 0.05; Fig. 6B). Due to the limited number of patients, the WS for Caspase-8 expression was arbitrarily dichotomized with a score of ≤ 6 being classified as “low” and > 6 as “high” expression. The median pCDK9 signal significantly increased in tumors with a low Caspase-8 expression (*p* = 0.004; Fig. 6C, Table 1).

**Table 1:** Correlation between CASP8 expression and pCDK9 level in cervical cancer patients

	No.	CASP8 low	CASP8 high	*p* value	No.	pCDK9 low	pCDK9 high	*p* value
		*n* (%)	*n* (%)			*n* (%)	*n* (%)	
CASP8								
Low (WS≤6)					42	19 (27.7)	23 (33.3)	0.018
High (WS>6)					27	20 (30.0)	7 (17.9)	
pCDK9								
Low (≤med)	39	19 (27.7)	20 (30.0)	0.018				
High (>med)	30	27 (39.1)	7 (10.3)					

*WS* Weighted Score, *med* median

To investigate whether the correlation between Caspase-8 expression and pCDK9 levels in cervical cancer patients also extended to phospho-RNAPII levels, we next knocked-down Caspase-8 expression in primary cells derived from a cervical tumor. Immunoblotting revealed enhanced pCDK9 and phospho-RNAPII in the siCasp8-treated cells compared to siCtrl transfected cells (Fig. 6D, bar graph). In addition, the knock-down of Caspase-8 expression in cervical cancer patient-derived cells also led to a significant increase in their invasiveness compared to non-transfected (NT) or siCtrl transfected cells (Fig. 6E).

In summary, these data validated that, in cervical cancer patients, low or high Caspase-8 expression significantly correlated with increased or decreased pCDK9 levels and, by extension, phospho-RNAPII levels, respectively. These results are concomitant with our cellular observations and strongly suggest that low Caspase-8 expression in cervical cancer patients might stimulate enhanced cell-migration and cell-invasion.

### Caspase-8 negatively regulates the transcription of cell-migration- or cell-invasion-associated genes

**Fig. 7:**
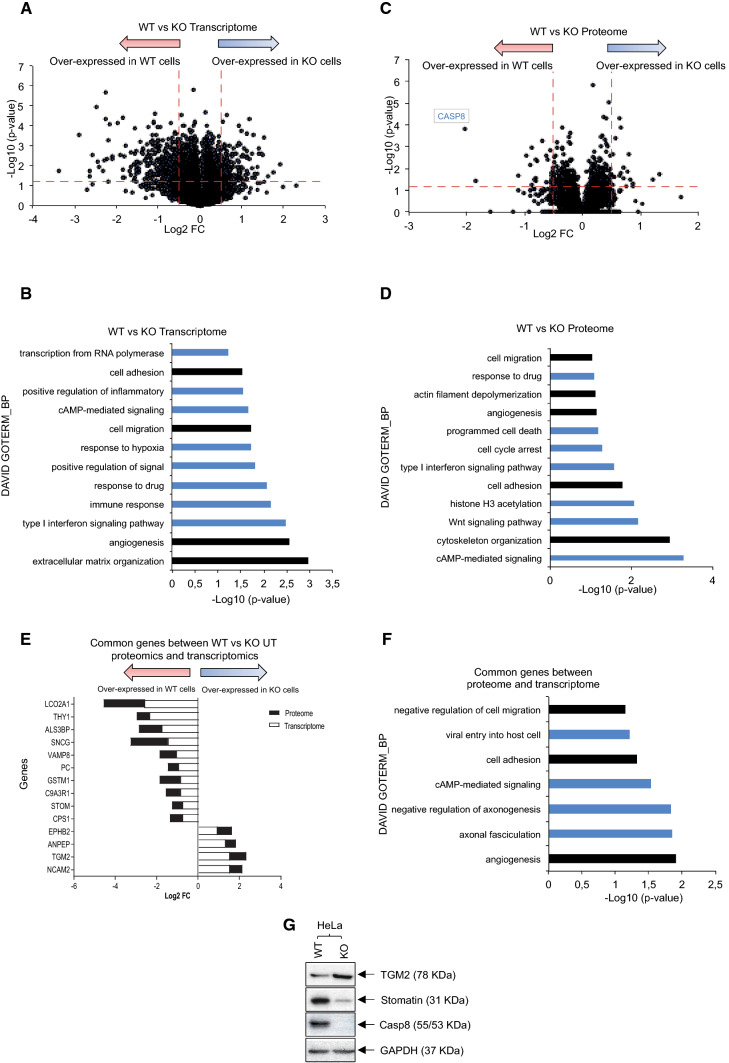
Identification of differentially expressed genes in the presence/absence of Caspase-8. **A** Comparison of differentially expressed transcripts in HeLa WT and KO cells, as detected through HumanHT-12 v3 microarrays. > 48,000 transcripts were detected when not adjusted for FDR *q* value. The red dashed lines represent a cut-off of − Log10 (*p* value) of ≤ 1.30 (y-axis) and Log2 FC [non-synchronized (NS) and S/G2-phase synchronized (synch.)] of ≥ 0.5 (over-expressed in the KO cells) and ≥ − 0.5 (over-expressed in the WT cells) (x-axis). Normalization of the raw data by adjusting for FDR *q* value of ≤ 0.05 resulted in 302 transcripts, with an over-expression of 78 transcripts in the KO and 224 in the WT cells. (B) The top 15 functions regulated by these 302 transcripts were determined using the DAVID GOTERM_BP analysis. Highlighted in black are the processes associated with cell-migration. **C** Comparison of proteins differentially expressed in Tandem Mass Tag (TMT) labeled HeLa WT and KO cells detected through MS analysis. > 5000 proteins were initially detected, of which 60 proteins (33 in WT and 27 in KO cells) passed the cut-offs of − Log10 (*p* value) of ≤ 1.30 and Log2 FC [non-synchronized (NS) and S/G2-phase synchronized (synch.)] of ≥  ± 0.5, as represented by the red dashed lines. **D** The top 15 functions regulated by these 60 proteins, as determined using the DAVID GOTERM_BP analysis. Highlighted in black are the processes associated with cell-migration. **E** Of the 302 genes from transcriptome analysis and 60 protein-encoding genes from the proteome analysis, 14 were shared between the two data sets. Representation of the expression profiles of the 14 common genes in the transcriptome and proteome data sets. **F** The top 10 functions are regulated by the 14 common genes, as determined using the DAVID GOTERM_BP analysis. Highlighted in black are the processes associated with cell-migration. Both the transcriptome and proteome analyses were performed in triplicate. **G** Immunoblot of HeLa WT and KO cells representing the protein levels of TGM2, Stomatin, Caspase-8, and GAPDH

Having demonstrated the altered regulation of Caspase-8 on RNAPII-mediated global transcription, our next question was whether this could lead to alterations in the transcription of genes associated with cell-migration and cell-invasion. This might explain the enhancements in these cellular behaviors in the Caspase-8 KO cells (Fig. 2F, G, Supplementary Figs. 1D, E). With this intent, we first performed a transcriptomics analysis of non-synchronized (NS) and S/G2-phase synchronized (synch.) WT and KO HeLa cells (Fig. 7A). After normalization, ~ 300 transcripts were obtained using the cut-offs of Log10 (*p* value) of ≤ 1.30 and Log2 FC of ≥  ± 0.5, whose expressions were altered either in the WT [non-synchronized (NS) and S/G2-phase synchronized (synch.)] or KO [non-synchronized (NS) and S/G2-phase synchronized (synch.)] cells. The DAVID GOTERM_BP analysis of the ~ 300 transcripts revealed several cell-migration associated functions (Fig. 7B, highlighted in black). To determine whether the differential mRNA expressions of these genes, due to the presence/absence of Caspase-8 expression, also translated to their protein expression, we also performed proteomics analysis of non-synchronized (NS) and S/G2-phase synchronized (synch.) WT and KO HeLa cells (Fig. 7C). Once again, using the cut-offs of Log10 (p-value) of ≤ 1.30 and Log2 FC [non-synchronized (NS) and S/G2-phase synchronized (synch.)] of ≥  ± 0.5, we identified 60 proteins whose DAVID GOTERM_BP analysis revealed several cell-migration associated functions (Fig. 7D, highlighted in black). Comparison of the 60 proteins and ~ 300 genes registered 14 common genes between the two data sets, of which 10, like *STOM*, *SLC9A3R1* (NHERF1), were similarly over-expressed in the WT and 4, like *TGM2* and *EPHB2*, in the KO cells (Fig. 7E), whose DAVID GOTERM_BP analysis again revealed several cell-migration associated functions (Fig. 7F, highlighted in black).

To confirm our findings, we checked the protein expressions of two of these genes—*STOM* and *TGM2* in HeLa WT and KO cells due to their reported roles in promoting metastasis in different cancer entities [65, 66]. We observed that TGM2 expression was up-regulated, while Stomatin was down-regulated in the KO cells (Fig. 7G), precisely matching our analysis (Fig. 7E). Finally, the knock-down of TGM2 (siTGM2) in HeLa and SiHa WT and KO cells significantly reduced their 2D migration, as compared to their respective siCtrl transfected counterparts. This suggested that the enhanced expression of TGM2 is, at least in part, responsible for the enhanced migration of cervical cancer cells, lacking Caspase-8 expression (Supplementary Figs. 7A, B).

This finding is crucial as we could demonstrate that when the HeLa WT and KO cells were treated with increasing concentrations of the classic Epithelial to Mesenchymal Transition (EMT) inducing agent—TGF-β1 [67], the HeLa KO cells displayed significantly enhanced protein and mRNA levels of the mesenchymal marker—Vimentin [68] (Supplementary Figs. 8A, B) and significantly reduced mRNA levels of the epithelial marker—E-Cadherin [68] (Supplementary Fig. 8B), as compared to untreated HeLa WT cells.

In summary, through our transcriptomics and proteomics analysis, we identified multiple genes whose differential expressions in WT and KO HeLa cells could promote the enhanced cell-migration of the KO cells. The knock-down of one of these genes—*TGM2*, whose expression was up-regulated in the KO cells, significantly reversed the 2D cell-migration of both the HeLa and SiHa KO cells. This could be a factor in the enhanced EMT of the KO cells when stimulated with TGF-β1.

### The loss of Caspase-8 expression reduces the sensitivity toward the small-molecule CDK9 inhibitor BAY1251152 in cervical cancer

Enhanced CDK9 activity is detected in several cancer entities, resulting in increased transcription of cancer-promoting genes [38]. We have, thus far, demonstrated that low Caspase-8 expression in cervical cancer cell lines and primary tumors leads to higher CDK9 kinase activity. Therefore, our next objective was to determine whether any correlation between low Caspase-8 expression and sensitivity to small-molecule CDK9 inhibitors exists.

**Fig. 8:**
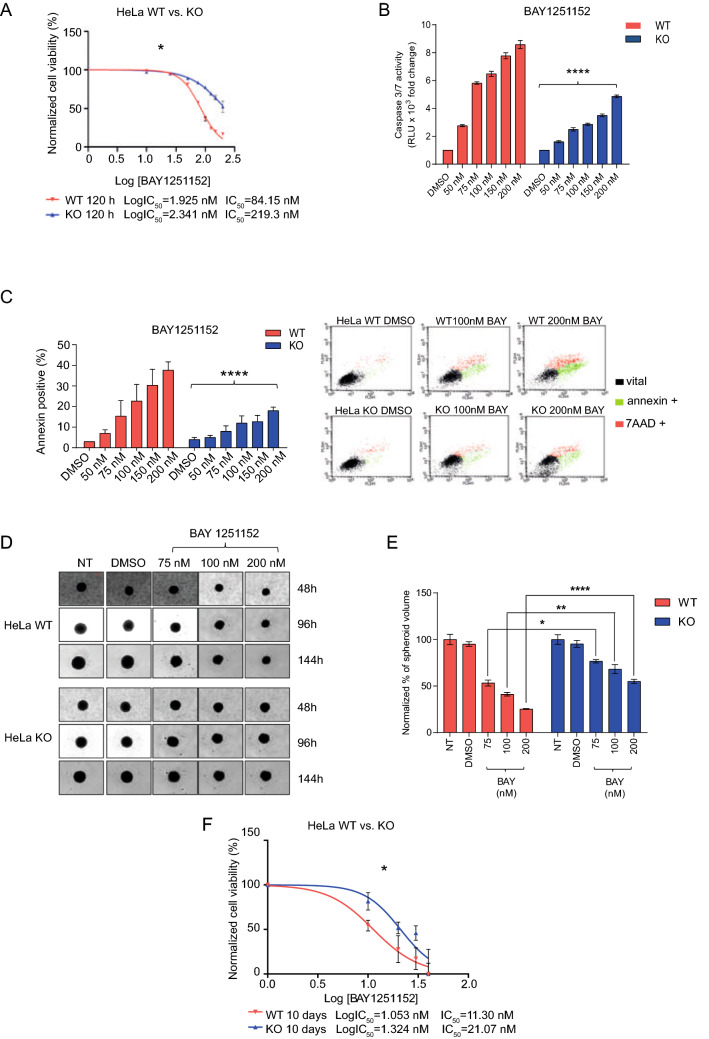
Effects of Caspase-8 knock-out on small-molecule inhibitor of CDK9. **A** HeLa WT and KO cells were treated with increasing concentrations of the small-molecule CDK9 inhibitor BAY1251152, and their proliferation was measured using an CellTiter-Blue Cell Viability assay over a period of 120 h, with measurements taken every 24 h. The proliferation rates of the treated cells, an indication of cell-viability, were normalized with their respective DMSO-treated counterparts (vehicle control = 0 on the x-axis) and subsequently used to calculate the IC50 values of each cell type. The plots represent the IC50 values at the 120 h time point. HeLa WT and KO cells were similarly treated for 48 h with increasing concentrations of BAY1251152 and used for **B** The measurement of Caspase-3/7 activities using a Caspase-Glo 3/7 assay normalized to their respective DMSO controls; and **C** Graphical representation of Annexin V positive (%) cells normalized to their respective DMSO controls. In the right panel, the FACS dot-plots of the WT and KO cells, gated for Annexin V/7AAD negative (vital), Annexin V positive (early apoptotic), and Annexin V/7AAD positive (late apoptosis) populations have been demonstrated. [for both **B** and **C**, mean ± SD; *n* = 3 for each concentration; *p* value (two-way Anova); **** =  < 0.0001]. 3D-spheroids of HeLa WT and KO cells were treated with increasing concentrations of BAY1251152 (75, 100, 200 nM). Untreated (UT) and DMSO-treated (vehicle control) cells were also included. (D) The left panel depicts the brightfield images of the HeLa WT and KO cell-derived spheroids at these time points (Scale bar = 200 µM); **E** The spheroid volumes following BAY1251152 and DMSO treatments were normalized to those of the UT spheroids, at each time point. Only the 144 h data is graphically represented here. The volumes of the spheroids were quantified by first measuring their area at 48, 96, and 144 h (after treatment) using the ImageJ program, which was then used to measure their radius. The radius of the spheroids was then used to measure their volumes. [*n* = 3 for each treatment; mean ± SD; *p* value (two-way Anova); * =  < 0.05; ** =  < 0.01; **** =  < 0.0001]. **F** Effect on the 3D colony-formation following the treatment of HeLa WT or KO cells with increasing concentrations of BAY1251152. 10 days after plating, the colonies were counted microscopically from 3 individual experiments, normalized with their DMSO (0 on x-axis) controls, and subsequently used to calculate the IC50 values of each cell type [*n* = 3; for all IC50 experiments, mean ± SD; p-value (paired *t* test, two-tailed); * =  < 0.05]

For this, we treated the HeLa WT and KO cells with increasing concentrations of the CDK9 inhibitor BAY1251152 [38] and determined their proliferation over 120 h (Fig. 8A). HeLa KO cells were significantly less sensitive to BAY1251152 than their WT counterpart (IC50 WT vs. KO: 84.15 vs. 219.3 nM). BAY1251152 also induced significantly lower levels of apoptosis in KO cells, as measured by Caspase-3/7 activities (Fig. 8B) and Annexin V/7AAD staining (Fig. 8C). This suggests that the absence of Caspase-8 expression de-sensitizes cervical cancer cells to CDK9 inhibitors.

To rule out the possibility that the loss of sensitivity of the KO cells to the CDK9 inhibition is not due to the loss of the apoptotic function of Caspase-8, we rescued the KO-cells with either Flag-Casp8-WT or its catalytically inactive mutant (Flag-Casp8-C360A) [69], and subjected them to BAY1251152 treatment (Supplementary Fig. 9A, B). We found that the expression of both Flag-Casp8-WT and -C360A comparably sensitized the KO cells to BAY1251152 treatment as compared to the Empty Flag-Vector (EV, negative control) transfected cells, as indicated by the increase in PARP cleavage (Supplementary Fig. 9A) and the enhanced activation of Caspase 3/7 (Supplementary Fig. 9B). No appreciable decrease in Flag-Casp8 levels (WT or C360A) was observed in BAY1251152 vs. DMSO treated cells (Supplementary Fig. 9A), indicating the absence of Caspase-8 cleavage upon CDK9 inhibition in the rescued cells. The data confirm that the re-sensitization of the KO cells to CDK9 inhibition occurs, in this context, irrespective of the apoptotic function of Caspase-8.

We next wanted to investigate the long-term efficacy of BAY1251152 on the 3D-growth of cervical cancer cell lines, which mimic the physiological conditions more accurately [70, 71]. Long-term treatment (up to 144 h) of the 3D-spheroids, derived from HeLa WT and KO cells, with increasing concentrations of BAY1251152, showed that the lack of Caspase-8 expression significantly attenuated the response of HeLa KO cells to CDK9 inhibition. Accordingly, BAY1251152 reduced the growth/volumes of the 3D-spheroids derived from HeLa KO cells significantly less than those derived from WT cells (down to 25% for the WT spheroids as compared to only 60% for the KO spheroids using 200 nM BAY1251152) (Fig. 8D, E). Additionally, we performed a 3D colony-formation assay on HeLa WT and KO cells treated with BAY1251152 for up to 10 days. The calculated IC50 values of BAY1251152 further confirmed the loss of sensitivity of the Hela KO-derived colonies as compared to their WT-derived counterparts (IC50: 11.30 vs. 21.07 nM, respectively) (Fig. 8F). These results are consistent with our previous findings, which indicated that the loss of Caspase-8 causes a cellular over-activity of CDK9 and highlights the critical role of Caspase-8 in improving the response and sensitivity of cervical cancer to CDK9 inhibition.

### Combinations of Cisplatin with BAY1251152 synergistically inhibit the 2D cell-proliferation and 3D clonogenic-growth of cervical cancer cells

The gold standard of cervical cancer treatment includes Carboplatin or Cisplatin in combination with radiotherapy. However, cervical cancers frequently develop resistance to platin-based chemotherapeutics [72, 73], severely undermining their treatment. Thus, we wondered whether targeting CDK9 would improve the response to Cisplatin-based chemotherapy in Caspase-8 KO cervical cancer cells.

**Fig. 9:**
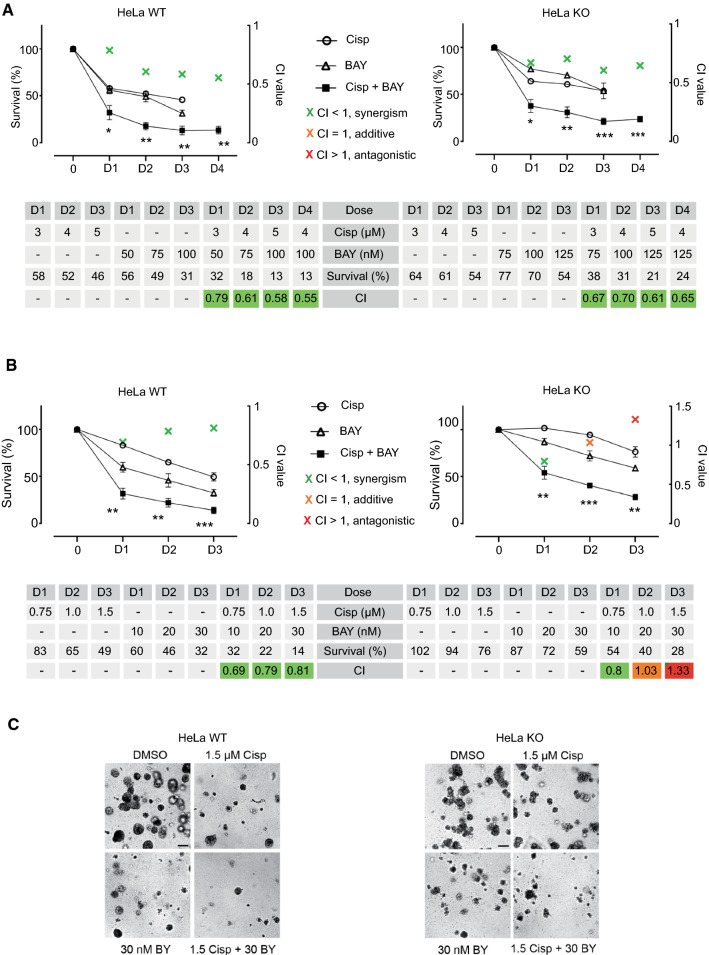
Effects of combination therapy of Cisplatin and BAY1251152. **A** HeLa WT and KO cells were treated with increasing concentrations of Cisplatin and BAY1251152, either alone or in combination (dose), and the 2D cell-proliferation was measured using an CellTiter-Blue Cell Viability, 120 h post-treatment. **B** HeLa WT and KO cells were treated with increasing concentrations of Cisplatin and BAY1251152, either alone or in combination (dose), and their 3D clonogenic-growth was measured. 10 days after plating, the colonies were counted microscopically from 3 individual experiments. The Combination Indices (CI), representing the integrated effects of these doses on cell survival, were calculated using the CompuSyn software. The doses, % of survival, and CI values for each cell line have been shown in their respective adjacent tables. (*n* = 3; 0 = DMSO vehicle control; all values represent mean ± SEM). [*p* values (paired *t* test, two-tailed; combinations vs Cisplatin alone); * =  < 0.05; ** =  < 0.01; *** =  < 0.001; CI values > 1 = antagonistic; = 1 = additive; and < 1 = synergistic]. **C** Representative images show the 3D colonies of HeLa WT and KO cells on day 10. (Scale bar = 100 µM)

For this purpose, HeLa WT and KO cells were treated with increasing concentrations of BAY1251152 or Cisplatin as single agents or in combination, and their 2D cell-proliferation was assessed over 120 h. Expectedly, the sensitive Caspase-8 WT cells reacted to the BAY1251152/Cisplatin combination by demonstrating significantly enhanced growth inhibition compared to the single treatments at all indicated concentrations. Furthermore, Combination Indices (CI) revealed that BAY1251152 synergistically enhanced the growth inhibitory effect of Cisplatin at all combinations tested (Fig. 9A). Remarkably, even the resistant Caspase-8 KO cells responded to BAY1251152/Cisplatin combination by also demonstrating significantly reduced survival, as compared to the single treatments, at all indicated concentrations. CI values once again revealed that BAY1251152 significantly and synergistically enhanced the growth inhibitory effect of Cisplatin at all combinations tested (Fig. 9A).

We also similarly treated HeLa WT and KO cells with BAY1251152 or Cisplatin as single agents or in combination and assessed their 3D clonogenic-growth over 10 days. As before, the BAY1251152/Cisplatin combination significantly decreased the clonogenic-growth of HeLa KO cells, but only the lowest combination of Cisplatin with BAY1251152 did so synergistically (0.75 µM + 10 nM; CI 0.80), as the higher combinations were either additive (1.0 µM + 20 nM; CI 1.03) or antagonistic (1.5 µM + 30 nM; CI 1.33) (Fig. 9B, C). Here again, the HeLa WT cells demonstrated a significant and synergistic decrease in clonogenic-growth at all combinations tested, which was also reflected in their corresponding CI values (Fig. 9B, C).

In summary, the results of our 2D- and 3D-cellular experiments demonstrated that the response of cervical cancer to Cisplatin-based standard chemotherapy might significantly improve by concurrently blocking CDK9 activity using small-molecule inhibitors. This renders CDK9 a promising molecular target for treating cervical cancer patients with low Caspase-8 expression.

## Discussion

Concurrent chemoradiation (CCRT) with Cisplatin or Carboplatin alone or combined with Paclitaxel, Gemcitabine, and Vincristine is the standard treatment approach for late-stage cervical cancer [74]. However, cervical cancer frequently develops chemotherapeutic resistance, which along with metastases, lowers the 5-year survival rate to just 17% [3, 5]. Therefore, investigating the molecular mechanisms underlying cervical cancer metastasis, the root causes of treatment resistance, and discovering new prognostic and predictive biomarkers may help to develop novel therapeutic strategies for this lethal malignancy. In addition to its traditional function of initiating cell death, Caspase-8 is also implicated in several non-apoptotic processes in various cancers, including cell-cycle control, proliferation, migration, invasion, and angiogenesis [10, 24–31, 74]. Furthermore, by modulating the enzymatic activities of CDK9 and RNAPII, our current investigation added a novel and crucial role for Caspase-8 in mediating transcription elongation.

First, our meta-analysis revealed that Caspase-8 expression levels correlate with tumor development and clinical outcome (Fig. 1). We discovered that low Caspase-8 expression is associated with poor OS and PFS in cervical cancer patients. Furthermore, the loss of Caspase-8 expression promotes migration and invasion in patient-derived cervical cancer cells (Fig. 6E), suggesting a correlation between Caspase-8 expression and elevated cervical tumor malignancy and aggression. These results suggested that cervical cancer patients' Caspase-8 protein expression profile could be used as a predictive biomarker.

Additionally, concurrently with the patient data, transcriptome and proteomic profiling of Caspase-8-depleted cervical cancer cell lines (HeLa and SiHa) identified numerous genes with altered expression, including *STOM* and *TGM2*, which may boost the propensity for metastasis and invasion. In cells missing Caspase-8, TGM2 levels increased, whereas stomatin levels declined (Fig. 7G). This finding was intriguing because stomatin has been shown to prevent metastasis in NSCLC cells, and its downregulation during EMT stimulates metastasis and is associated with poor prognosis [64]. Contrarily, the up-regulation of TGM2 expression/activity has been linked to a greater incidence of metastasis in various cancer entities [75–77]. In light of these findings, we could show that the knock-down of TGM2 expression dramatically decreased the ability of cervical cancer cells lacking Caspase-8 expression to migrate. This strongly suggests that Caspase-8 plays a crucial role in undermining metastasis-promoting pathways in cervical cancer. Interestingly, Caspase-8’s anti-metastatic function in this context appears to be mediated by transcriptional regulation and, more importantly, irrespective of its role in death receptor-induced apoptosis.

However, the most intriguing aspect of our study is finding a previously unknown non-apoptotic function of Caspase-8 in cervical cancer as a negative regulator of CDK9 kinase activity in vitro. Indeed, interactome analyses and multiple IP experiments have shown that CDK9 and Caspase-8 interact in vitro. Interestingly, this interaction inhibits CDK9 activity, as demonstrated by the decline in CDK9 phosphorylation at Thr186. Excitingly, IP and cellular fractionation experiments confirmed that the physical association between caspase-8 and CDK9 takes place within the nucleus (Fig. 4J), even though Caspase-8 has previously been reported to be a cytoplasmic protein [10, 18] and even though it also exhibits a nuclear localization sequence (NLS) in its p10 domain [78]. This finding is in line with previous studies reporting the accumulation of Caspase-8 in the nucleus of cervical cancer cells with high-risk human papillomaviruses (HPVs) expressing E6 and E7 proteins such as HeLa cells [75–77]. The nuclear accumulation of Caspase-8 is due to its stimulated recruitment by the E6 oncoprotein, such that Caspase-8 exerts beneficial functions for the viral life cycle [77]. Here, the oncoprotein E6-Caspase-8 association appears to have little impact on promoting Caspase-8’s apoptotic process, most likely caused partly by Caspase-8’s changed cellular location [77].

CDK9 is primarily activated by forming a heterodimeric complex involving Cyclin T1 to form P-TEFb, although minor cyclins like Cyclins T2a and T2b can also perform this function [78]. While around 80% of CDK9 remains as P-TEFb, sequestered in the nucleus either as an inactive complex 7SK snRNP or an active complex with BRD4 [38, 79–83], the remaining ~ 20% of inactive CDK9 remains within the cytoplasm, bound to two chaperones—HSP70 and the HSP90/CDC37 complex, and undergoes cytoplasmic to nuclear shuttling [38, 62, 84, 85]. The phosphorylation of the CDK9 Thr186 residue, either through autophosphorylation or by CDK7 [86], is critical for its association with Cyclin T1 [38, 87] and its dissociation from the HSP90/CDC37 complex [88]. Based on this, our data suggest that Caspase-8 allosterically inhibits the activity of CDK9, as measured by its autophosphorylation. This would occur by multiple mechanisms like modifying the conformation of CDK9 to block the Thr186 site or the ATP binding site, the hinge region, or by competing directly with CDK7 to prevent it from phosphorylating CDK9. Interestingly, we have been able to confirm here that, under in vitro conditions, Caspase-8 binds to CDK9 directly (Fig. 4C). Furthermore, as our ex vivo experiments have demonstrated a nuclear interaction between Caspase-8 and CDK9, taken together, these experiments have convincingly established that these two proteins interact, within the nucleus, without the presence of any bridging molecules like BRD4 or members of the 7SK snRNP complex like—HEXIM1/2, LARP7 and MePCE [38].

In addition, the loss of Caspase-8 caused what we can describe as a hyperactive state of CDK9, resulting in the increased phosphorylation of key CDK9 targets. Thus, we showed that Caspase-8 depletion increased CDK9-mediated phosphorylation of crucial substrates like RNAPII and SPT5 in vitro and in cells. This increase in RNAPII activity consequently caused an alteration in cellular transcription, as demonstrated by the increase in EU and the alteration of the expression of several genes (Fig. 7E, G), notably those involved in metastasis and invasion. Similarly, our investigation of a cohort of primary cervical cancer patient-derived tissues also confirmed a significant correlation between high Caspase-8 expression and low CDK9 Thr186 phosphorylation, and low RNAPII Ser2 phosphorylation (Fig. 6A–D).

This novel mechanism of Caspase-8-dependent inhibition of CDK9 is a singular finding that differs entirely from what has been reported in the literature about caspase-mediated regulation of protein kinases [18, 20, 89]. More specifically, many kinases, particularly cell-cycle-associated ones, have been described to undergo caspase-dependent cleavage activation [89]. Accordingly, an increased CDK2 kinase activity has long been closely linked to apoptosis. Furthermore, this activation has been shown to be triggered by a caspase-dependent cleavage cascade, directly by triggering the cleavage of Cyclin A, creating a proteasome-resistant and kinase-active form of Cyclin A/CDK2 involved in the apoptotic response to ionizing radiation [90], and indirectly by inducing the cleavage of the CDK inhibitors, p21Cip1/Waf1, and p27Kip1 [91], or WEE1 inactivation [92], which promotes inhibitory phosphorylations of CDK2 during apoptosis, and consequently leading to increased activity of CDK2 [91].

One of the significant findings of our study can be summarized by the fact that in cervical cancer cell lines, as well as in patient-derived cells, loss of Caspase-8 induces an over-activation of CDK9, which consequently alters the cellular transcriptional landscape (transcriptional re-programming), by increasing RNAPII activity. This represents, most likely, one of the potential resistance mechanisms of advanced cervical cancer to standard chemotherapy, similar to what has been described in medulloblastoma resistance to conventional therapy [93].

This significant finding has given us a solid rationale for targeting low Caspase-8 expressing cells with increased CDK9 kinase activity, using small molecule CDK9 inhibitors, either alone (Fig. 8) or in combination with Cisplatin (Fig. 9). We could demonstrate that combining Cisplatin with the small-molecule CDK9 inhibitor—BAY1251152, could overcome the chemoresistance and restore the sensitivity of HeLa KO cells toward Cisplatin, synergistically and significantly, again under both 2D- and 3D-conditions (Fig. 9). Not surprisingly, the HeLa WT cells, with low CDK9 activity, were synergistically targeted by a combination of BAY1251152 and Cisplatin, under both growth conditions. This finding is important because several third-generation CDK9 inhibitors like BAY1251152, AZD-4573, and JSH-150 are already undergoing pre- or clinical trials [38], and newer strategies are being applied for the identification of novel inhibitors against transcription-associated CDKs like CDK9 [94]. Moreover, there are excellent precedents for combining standard chemotherapeutics with small-molecule inhibitors of CDKs like those against CDK 4/6 [95, 96].

Additionally, our observation that in cervical cancer patients with low TMB, low Caspase-8 expression elicits a significantly better prognosis than in patients with high Caspase-8 expression (Fig. 1B) could be explained by the possibility that during the early stages of cervical cancer development, localized cervical cancer cells are not overtly dependent on the altered expressions of metastasis inducing genes. Therefore, the presence of Caspase-8 in these cells is not a serious impediment to the development of this cancer. Only during invasive stages the expression of Caspase-8 is down-regulated to aid in the process. Indeed, it has been reported that TMB in cervical cancer significantly correlates with its stages [97]. The presence of Caspase-8 in the earlier stages probably also assists the chemotherapeutic treatment of this cancer, eliciting a better prognosis [16].

In conclusion, we believe that the data presented by this study support further pre-clinical and clinical evaluations of CDK9 inhibitors in advanced and resistant cervical cancer. However, a more rigorous assessment of Caspase-8 expression and the corresponding stratification of patients will be of utmost importance in determining the clinical success of the CDK9-based targeted strategies in cervical and other cancer entities in the future.

## Supplementary Information

Below is the link to the electronic supplementary material.

Supplementary file1 (EPS 7759 KB)

Supplementary file2 (EPS 1835 KB)

Supplementary file3 (EPS 3603 KB)

Supplementary file4 (EPS 3672 KB)

Supplementary file5 (EPS 2005 KB)

Supplementary file6 (EPS 2749 KB)

Supplementary file7 (EPS 3595 KB)

Supplementary file8 (EPS 1723 KB)

Supplementary file9 (EPS 1835 KB)

Supplementary file10 (DOCX 20 KB)

## Data Availability

All materials used in this work will be provided upon request. Illumina microarrays were deposited in the Gene Expression Omnibus (GEO) under the accession number GSE217360. The mass spectrometry proteomics data have been deposited to the ProteomeXchange Consortium via the PRoteomics IDEntifications Database (PRIDE) partner repository with the dataset identifier PXD038102.
