# Correction: The non-apoptotic function of Caspase-8 in negatively regulating the CDK9-mediated Ser2 phosphorylation of RNA polymerase II in cervical cancer

**DOI:** 10.1007/s00018-023-04686-y

**Published:** 2023-02-18

**Authors:** Ranadip Mandal, Monika Raab, Franz Rödel, Andrea Krämer, Izabela Kostova, Samuel Peña-Llopis, Gioele Medici, Björn Häupl, Thomas Oellerich, Khayal Gasimli, Mourad Sanhaji, Sven Becker, Klaus Strebhardt

**Affiliations:** 1 Department of Gynecology, University Hospital, Frankfurt am Main, Germany; 2 Department of Radiotherapy and Oncology, University Hospital, Frankfurt am Main, Germany; 3 German Cancer Consortium (DKTK), Frankfurt am Main, Germany; 4 Frankfurt Cancer Institute (FCI), Goethe University, Frankfurt am Main, Germany; 5 Translational Genomics in Solid Tumors, West German Cancer Center, University Hospital, Essen, Germany; 6 German Cancer Consortium (DKTK), Essen, Germany; 7 German Cancer Research Center (DKFZ), Heidelberg, Germany; 8 Laboratory of Molecular Neuro-Oncology, Department of Neurology & Brain Tumor Center, University Hospital Zurich, University of Zurich, Zurich, Switzerland; 9 Clinical Neuroscience Center and Department of Neurosurgery, University Hospital Zurich, University of Zurich, Zurich, Switzerland; 10 Medizinische Klinik 2-Haematology/Oncology, University Hospital, Frankfurt am Main, Germany

**Correction: Cellular and Molecular Life Sciences (2022) 79:597** 10.1007/s00018-022-04598-3

In the original version of this article, Gioele Medici was missing in the author group.

The updated author group is

Ranadip Mandal, Monika Raab, Franz Rödel, Andrea Krämer, Izabela Kostova, Samuel Peña-Llopis, Gioele Medici, Björn Häupl, Thomas Oellerich, Khayal Gasimli, Mourad Sanhaji, Sven Becker, Klaus Strebhardt

The original article has been updated.

