# Supplementary material for: The non-apoptotic function of Caspase-8 in negatively regulating the CDK9-mediated Ser2 phosphorylation of RNA polymerase II in cervical cancer
Source: Cell Mol Life Sci. 2022 Nov 18;79(12):597. doi: 10.1007/s00018-022-04598-3 (PMC9674771; doi:10.1007/s00018-022-04598-3)
Supplement: Supplementary file 10 — Supplementary file10 (DOCX 20 KB) [file 18_2022_4598_MOESM10_ESM.docx]

**Supplementary Figure Legends**

**Supplementary Figure 1: Effects of the knock-out of Caspase-8 on the behavior of cervical cancer cell lines. (A)**Immunoblotting of SiHa CASP8-/- knock-out clones, generated with the CRISPR/Cas9 genome editing system, using antibodies against Caspase-8 and GAPDH. **(B)** SiHa WT and KO cells were synchronized with double-thymidine treatment for 16 h and then released for 0, 3, 6, and 9 h. Cells were harvested, treated with Propidium Iodide (PI), and analyzed by FACS to reveal their cell-cycle distribution at each time point. Non-synchronized (NS) WT and KO cells were used as negative controls. Overlay of the histograms of PI-positive WT and KO cells at every time point has been displayed in the lower panel.**(C)**Cells were lysed and immunoblotted to check for the cell-cycle markers PLK1, Cyclins B1, E1, and A1, as well as Caspase-8 and β-Actin. **(D)**The migration of SiHa WT and KO cells was determined using ibidi migration chambers at 2 h intervals over 30 h. The reduction in the areas between the two cell populations at each time point, representing the migration of cells, was measured, normalized to the area at 0 h, and represented graphically [mean ± SD; n = 3 for each time point; p-value (paired t-test, two-tailed); **** = < 0.0001]. **(E)**The 3D invasion of SiHa WT and KO cells using Matrigel coated invasion chambers was determined over 24 h. The nuclei of the invaded cells were stained with DAPI (bottom panel) and the quantification represented graphically [mean ± SD; n = 3 for each time point; p-value (paired t-test, two-tailed); ** = < 0.005].

**Supplementary Figure 2: Effects of the knock-out of Caspase-8 on the behavior of individual knock-out clones. (A)** The proliferation of three individual KO clones of HeLa (K5, 7, and 8) was measured using an MTT assay. Over 120 h, the number of viable cells was quantified every 24 h and represented graphically [mean ± SD; n = 3 for each time point; p-value (paired t-test, two-tailed); n.s. = not significant].**(B)**The 2D migrations of the three individual KO clones of HeLa and SiHa (K7, 11, and 22) were determined using ibidi migration chambers at 3 h intervals over 24 h. The reductions in the areas between the two cell populations at each time point, representing the migration of the cells, were measured, normalized to the area at 0 h, and represented graphically [mean ± SD; n = 3 for each time point; p-value (paired t-test, two-tailed); n.s. = not significant].

**Supplementary Figure 3: Effects of the knock-out of Caspase-8 on the behavior of cervical cancer cell lines. (A)**Endogenous Caspase-8 in HeLa and SiHa WT cells was transiently knocked down with siCasp8, or Flag-Casp8 was over-expressed in HeLa WT and KO cells. For HeLa WT cells, Empty Flag-Vector (EV) and siCtrl-transfected; for HeLa KO cells, Empty Flag-Vector (EV); and for SiHa WT and KO cells, non-transfected (NT) and Empty Flag-Vector (EV) controls were included. Immunoblots of both cell lines were probed for Flag-Casp8, Caspase-8, and β-Actin or GAPDH to check for the efficiency of the transfections. **(B)**These cells were then subjected to 3D cell-invasion assays over 24 h. The graphs represent the quantification of the DAPI-stained invaded cells, and the bottom panels show the nuclear staining of the invaded cells with DAPI (mean ± SD; n = 3 for each cell type). **(C)**For a 2D cell-migration assay of HeLa cells, over a period of 24 h, the reduction in the areas between the two cell populations, at 2 h intervals, representing the migrations of the cells, was measured, normalized to the area at 0 h, and represented graphically [for **B and C,**mean ± SD; n = 3 for each time point; p-value (paired t-test, two-tailed); * = < 0.05; ** = < 0.005; *** = 0.001; n.s. = not significant]. **(D)** HeLa WT and KO cells were treated with Trail and Cycloheximide (CHX) for 0.5, 1, and 2 h and subjected to Annexin V/7AAD apoptosis analysis. The graph shows the % of Annexin positive cells representing early apoptosis at each time point. The immunoblot was probed for Caspase-8 and GAPDH to determine Caspase-8 cleavage (0 h = untreated control; mean ± SD; n = 3).

**Supplementary Figure 4: Caspase-8 interactome. (A)**Graphical representation of the ratios of the Log2 FC expression values of the ~1000 proteins detected in the non-synchronized (NS) and ~850 proteins detected in the S/G2-phase synchronized (synch.) data-sets of HeLa WT cells in our interactome analysis. These proteins were common but differentially expressed in the non-synchronized (NS) and S/G2-phase synchronized (synch.) data sets. The red-dashed lines represent a Log2 FC cut-off of ≥ ± 0.5 (up- and down-regulated). Caspase-8 was also detected and has been marked in the graph. 551 proteins, which passed the cut-off (outside the dashed lines), were selected for further analysis. **(B)**Graphical representation of the expression profiles of the 551 proteins with Log2 FC of ≥ ± 0.5 (up- and down-regulated). These proteins were common but differentially expressed in both the non-synchronized (NS) and S/G2-phase synchronized (synch.) data sets of HeLa WT cells. **(C)**Graphical representation of 291 Caspase-8 interacting proteins, with a Log2 FC of ≥ + 0.5 (up-regulated), which were amongst the 551 proteins, commonly expressed between the non-synchronized (NS) and S/G2-phase synchronized (synch.) data sets of HeLa WT cells. The expressions of Caspase-8 and CDK9 have been shown in this graph. **(D)**Schematic representation depicting the stepwise selection of the 291 proteins, shown in **(C)**. Initially, the interactome analysis of Caspase-8 in HeLa WT the non-synchronized (NS) and S/G2-phase synchronized (synch.) data sets revealed ~6200 proteins, which were eventually narrowed down, using a Log2 FC cut-off of ≥ ± 0.5, to ~1000 proteins in the NS and ~850 proteins in the synch. Data-sets. 551 proteins were commonly expressed between the non-synchronized (NS) and S/G2-phase synchronized (synch.) data sets, with a Log2 FC of ≥ ± 0.5 (up- and down-regulated). Finally, 291 proteins were selected, with a Log2 FC cut-off of ≥ + 0.5 (up-regulated), which represented the pool of Caspase-8 interacting proteins. **(E)** Cell-migration-associated proteins from the non-synchronized (NS) and S/G2-phase synchronized (synch.) data sets, as predicted by DAVID in **Fig. 3C**, were found to form a tight cluster by the STRING bioinformatics tool (https://string-db.org/, v.11.0).

**Supplementary Figure 5: Quantitation of phosphorylation signals.**Phosphorylation signals in the presence of increasing amounts of GST-Caspase-8, shown in in vitro kinase assays in **Figure 5**, were quantified and normalized to the phosphorylation signals of the respective GST amounts for**(A)**pCDK9;**(B)**pCTD; and**(C)**pSPT5.

**Supplementary Figure 6: Effect of Caspase-8 on the autophosphorylation and enzymatic activity of CDK9. (A)**An anti-Cyclin T1 antibody was used to IP Cyclin T and active CDK9 from the lysates of HeLa WT and two knock-out clones (K5, K7). Precipitated Cyclin T1 and CDK9 were then incubated with non-radioactive ATP, His-CTD, and immunoblotted. Lysate input probed for pPOLR2A, POLR2A, CDK9, Caspase-8, and β-Actin (upper panel), and immunoblot of the Cyclin T1 IP, which was probed for pHis-CTD (with an anti-pPOLR2A antibody), CDK9, Cyclin T1, and unphosphorylated His-CTD (with an anti-His antibody) (lower panel). **(B)**The cytoplasm and nucleus of SiHa WT and KO cells were fractionated and used for immunoblotting with antibodies for pCDK9, CDK9, Cyclin T1, the transcription regulators - BRD4, NELF-A, and SPT5, pPOLR2A, POLR2A, Caspase-8, β-Actin, GAPDH (cytosolic marker) and Histone H3 (nuclear marker). **(C)**An EU assay was performed using Empty Flag-Vector (EV) transfected and 0.5 or 1.0 µg Flag-Casp8 transfected HeLa KO cells. The EU to DAPI stain fluorescence intensity ratio was quantified, normalized to Empty Flag-Vector (EV) transfected cells, and graphically represented. **(D)**EU and DAPI staining of HeLa WT siCtrl and 6.25 or 12.5 nM siCasp8-transfected cells. The ratio of fluorescence intensity of EU to DAPI stain was quantified, normalized to Empty Flag-Vector (EV) transfected cells, and represented graphically [for **C and D,** mean ± SD; n = 3 for each cell type; p-value (paired t-test, two-tailed); * = < 0.05; ** = < 0.005; **** = < 0.0001].

**Supplementary Figure 7: Effect of TGM2 expression on the 2D cell-migration, in the presence or absence of Caspase-8 expression, of cervical cancer cells. (A)**WT and KO cells were transfected with 25 nM siTGM2 for 24 h, and they were harvested and re-seeded in ibidi migration chambers and allowed to settle down for a further 24 h. Chambers were removed, and cells were allowed to migrate towards each other for 22 h, with cell-migration recorded every 2 h. siCtrl transfected WT and KO HeLa cells were used as controls for their respective siTGM2 transfected counterparts. The time-dependent decrease in the areas between the two sides of the chambers, normalized to the area at 0 h, has been represented graphically (upper panel). Progress of cell-migration at the 0 and 10 h time points for each cell type (lower panel). **(B)**42 h for the SiHa cells, with cell-migration, recorded every 6 h. siCtrl transfected WT and KO SiHa cells were used as controls for their respective siTGM2 transfected counterparts. The time-dependent decrease in the areas between the two sides of the chambers, normalized to the area at 0 h, has been represented graphically (upper panel). Progress of migration at the 0 and 42 h time points, for each cell-type (lower panel) [n = 3 for each time-point; mean ± SD; p-value (paired t-test, two-tailed), * = < 0.05; ** = < 0.005; *** = < 0.001; **** = <0.0001; * = WT siCtrl vs WT siTGM2; * = KO siCtrl vs KO siTGM2; * = WT siCtrl vs KO siCtrl; * = WT siTGM2 vs KO siTGM2].

**Supplementary Figure 8: EMT markers in the presence of TGF-β1. (A)**HeLa WT and KO cells were treated with increasing concentrations of TGF-β1 for 48 hours. The cells were then immunoblotted and probed for Caspase-8, Vimentin (Mesenchymal marker), and GAPDH. **(B)**HeLa WT and KO cells were treated for 48 hours with 10 ng/ml of TGF-β1. The mRNA expression levels of Vimentin and the Epithelial marker – E-Cadherin, were determined through qRT-PCR from their cDNAs [n = 3 for each cell-type; mean ± SD; p-value (Student’s t-test), * = < 0.05; ** = < 0.005; *** = < 0.001; n.s. = not significant].

**Supplementary Figure 9: Sensitivity of cervical cancer cells to BAY1251152** **in the presence of catalytically active or inactive Caspase-8 mutants.**HeLa KO cells were either transfected with Flag-Casp8 WT or -Casp8 C360A (catalytically inactive mutant). Empty Flag-Vector (EV) KO cells were used as a negative control, while HeLa WT cells were used for comparison. All cell types were treated with BAY1251152 (100 or 150 nM) for 48 h. DMSO treatment (vehicle control) was also included. **(A)**The Western blot was checked for the cleavages of PARP and Caspase-8 (both endogenous and Flag-Casp8). β-Actin was the loading control. **(B)**Graphical representation of Caspase-3/7 activities (fold change) following each treatment, normalized to their respective DMSO controls [n = 3 for every treatment; mean ± SD; p-value (two-way Anova); * = treatments vs. DMSO control; * = < 0.05; n.s. = not significant].
